# Direction of impact for explainable risk assessment modeling

**DOI:** 10.1111/risa.70003

**Published:** 2025-02-25

**Authors:** Emanuele Borgonovo, Manel Baucells, Antonio De Rosa, Elmar Plischke, John Barr, Herschel Rabitz

**Affiliations:** ^1^ Bocconi University Milan Italy; ^2^ Darden Business School University of Virginia Charlottesville Virginia USA; ^3^ Institute of Resource Ecology Helmholtz‐Zentrum Dresden Rossendorf Dresden Germany; ^4^ Department of Chemistry Princeton University Princeton New Jersey USA

**Keywords:** artificial intelligence, convexity, graphical visualization, machine learning, monotonicity, risk analysis, sensitivity analysis

## Abstract

Several graphical indicators have been recently introduced to help analysts visualize the marginal effects of inputs in complex models. The insights derived from such tools may help decision‐makers and risk analysts in designing interventions. However, we know little about the adequacy and consistency of different indicators. This work investigates popular marginal effect indicators to understand whether they yield indications consistent with the properties of the quantitative model under inspection. Specifically, we examine the notions of monotonicity, Lipschitz, and concavity consistency. Surprisingly, only PD functions satisfy all these notions of consistency. However, when selecting the indicators, in addition to consistency, analysts need to consider the risk of model extrapolation. For situations where such risk is under control, we utilize individual conditional expectations together with PD plots. Two applications, on a NASA space risk assessment model and a susceptible exposed infected recovered (SEIR) model for the COVID‐19 pandemic illustrate the insights obtained from these indicators.

## 1 INTRODUCTION

Risk assessment investigations are often supported by the creation of quantitative models. The data‐driven and AI revolutions have enriched the palette of available tools that nowadays range from agent‐based simulators (A. C. Reilly et al., 2021; Tonn & Guikema, 2018; Zhai et al., 2023), to system dynamics (Korzebor & Nahavandi, 2024; Logrosa et al., 2022), and to machine learning approaches (Agarwal et al., 2020; Aziz et al., 2023; Yao et al., 2024; Zhuang et al., 2022). With the increased sophistication of these models, we have a pressing need for methods capable of making the input–output black box more transparent.

Recent literature shows a confluence between the problem of opening the input–output black box and the tasks of sensitivity analysis. Reviews such as Guidotti (2018) and Murdoch et al. (2019a) highlight shared goals such as determining feature importance, the direction of impact, and the relevance of interactions. The recent position papers of Razavi (2021) and Scholbeck (2023) also emphasize these connections.

One key insight to enhance model transparency is understanding how the risk metric depends marginally on relevant inputs. In a seminal article regarding numerical computer experiments, Persi Diaconis provocatively asks what it means to know a function (Diaconis, 1989, p. 163). In answering, Diaconis underlines the need to know facts such as whether it is *monotone,…, convex, etc*. (Diaconis, 1989, p. 163). Understanding these properties requires studying the marginal behavior of the model output as a function of the inputs. In sensitivity analysis, this question falls within the direction of impact setting. (A setting is the up‐front formulation of the sensitivity analysis question aimed at helping analysts frame the investigation; Saltelli, 2002). Knowing these facts allows analysts to increase their awareness of the model response. Moreover, they can answer specific questions. Determining whether the risk metric increases or decreases or is concave/convex in the parameters is an important element in informing decisions and formulating interventions. Convexity is a key property in risk assessment, with applications ranging from the study of risk attitudes (Wakker & Yang, 2021), to the characterization of dose–response curves in carcinogenic risk studies (L. A. Cox, 1995; Crump, 1998; French & Williams, 2001; Spassova, 2019).

In a direction of impact setting, analysts need tools that can communicate straightforwardly and consistently the relevant insights to the stakeholders. Popular methods include tornado diagrams, spiderplots, and one‐way sensitivity plots (Bhattacharjya & Shachter, 2008; R. A, Howard, 1988). These tools often rely on a hidden monotonicity assumption. If this assumption holds, the values obtained when the inputs are at their extrema would provide upper and lower bounds to the output variability. However, this is not the case when the model is complex and interactions between inputs emerge. In these cases, we need graphical indicators that cope with probabilistic inputs, non‐monotonic behavior, and interactions.

Machine learning research has developed new graphical indicators to address these challenges. Partial dependence (PD) plots (Friedman, 2001), individual conditional expectation (ICE) plots (Goldstein et al., 2015), and accumulated local effect (ALE) plots (Apley & Zhu, 2020) are becoming widely employed. These tools take input distributions into account. As a drawback, they provide average indications which may not hold on a case‐by‐case basis. This contrasts with spiderplots, tornado diagrams, and one‐way sensitivity plots whose indications are local, with a specific base‐case in mind. Exploring the literature, analysts would also find a wider set of indicators comprising traditional gradient‐based methods (Iman & Helton, 1988; Tsanakas & Millossovich, 2016) as well as familiar statistical tools such as correlation coefficients (Ellouze et al., 2010).

Under uncertainty, analysts may regard the sign of the correlation coefficient between the risk metric and the variable of interest as an appropriate indicator. However, the indication might conflict with the information delivered by (say) a spiderplot calculated for the same model. Clearly, the assertion that the insights differ because a spiderplot is a local method, while a correlation coefficient provides an average insight is not enough. This raises the questions: Can insights from alternative indicators be reconciled? And, if not which one should analysts trust? While all these methods seek an answer to the same question, the extent to which they do so consistently is unclear.

Our purpose is to fill in this gap by proposing a systematic investigation of the properties of the direction of impact indicators. To do so, we employ the notion of consistency: It is desirable that graphical indicators yield insights in agreement with the properties of the mapping that links the quantity of interest to its inputs. We perform a thorough analysis considering relevant mathematical aspects in describing the nature of input–output mappings, such as convexity, monotonicity, and Lipschitz continuity. We also consider the multiplicative and additive recovery properties.

We investigate which indicators remain consistent not only when inputs are uncertain but also when they are correlated.

We study local and global indicators. As local indicators, we analyze differentiation‐based indices and one‐way sensitivity functions. As global indicators, we study correlation coefficients, partial derivatives, one‐way sensitivity functions, conditional expectation, PD, and ALE functions. We provide mathematical proofs or counterexamples about whether they are consistent with relevant properties of the quantity of interest such as monotonicity, Lipschitz continuity, and convexity, both with dependent and independent inputs.

We find (unsurprisingly) that local indicators are consistent with independent and dependent inputs. In contrast, among global indicators, (surprisingly) only PD functions are consistent with the above‐mentioned properties of the quantity of interest, both with dependent and independent inputs.

For all the listed indicators, we find closed‐form expressions for the class of affine input–output mappings. This family encompasses the structure of several models that support quantitative risk assessment, such as Bayesian networks, fault trees, and event trees, which are frequently used in risk analysis.

Our results underscore some of the theoretical properties of marginal behavior indicators. However, before a selection can be made, analysts need to consider extrapolation risk. Extrapolation errors may occur if a model is forced to predict in regions far from those on which it has been trained or for which it was designed. We discuss how to integrate consideration of extrapolation issues with our theoretical findings in the final remarks.

We present two applications of the findings. The first is the NASA probabilistic safety assessment for the analysis of lunar space missions. The second is a case study involving an epidemiological model fitted to data from the first phase of the COVID‐19 pandemic. The resulting modeling insights and risk management implications are discussed.

## 2 RELATED LITERATURE

This section is divided into two parts. In the first part, we review the literature on the intersection between quantitative modeling and sensitivity analysis in risk assessment studies. In the second part, we focus on graphical indicators in the direction of impact setting.

### 2.1 Quantitative modeling and sensitivity analysis in risk analysis

Quantitative modeling has a long tradition in risk analysis (Apostolakis, 1990; D. C. Cox & Baybutt, 1981). Applications range from studying the safety of nuclear reactors (Apostolakis, 2004), of radioactive waste repositories (Helton & Davis, 2002), to climate change and environmental risk assessment (Martellon et al., 2024; Tonn et al., 2020). With the data‐driven revolution and the expansion in computing power, risk analysts are increasingly relying on the use of machine learning and artificial intelligence methods. Gao and Wang (2023) adopt an explainable machine learning approach to help in planning hurricane responses, and Aziz et al. (2023) compare the efficacy of nine machine learning models in the risk assessment of immigration flows. Karanth and Pradhan (2023) introduce a machine learning approach for estimating the microbial dose response to salmonella exposure including genetic information, with such estimation playing an important role in food safety assessment. Yao et al. (2024) use an interpretable machine learning model based on the extreme‐gradient boosting techniques to assist in the assessment of arctic navigation risks. Thekdi et al. (2023) develop a framework for the use of artificial intelligence in disaster risk assessment and management. Such a rapid surge in the use of machine learning models exposes us to the perils of a mechanical application of tools in an *absence of theory* (Begoli et al., 2019) mode. Recent works recognize the importance of increasing the interpretability of machine learning models and of offering in‐depth explanations of their findings (Guidotti, 2018; Murdoch et al., 2019b; Rudin, 2019, 2022). When post hoc explanations are of concern, a recent position paper by Scholbeck (2023) highlights that several of the associated tasks are, in fact, part of what in risk analysis is referred to as sensitivity analysis.

Sensitivity and uncertainty analysis have integrated risk modeling since its start. A review of methods is already presented in D. C. Cox and Baybutt (1981), in the very first year of the *Risk Analysis* journal. This work is followed by several others, such as Iman (1987), Iman and Helton (1991), and Iman et al. (2005). Saltelli (2002) takes a broad perspective and considers the problem of defining up front the question that the analyst must address with the sensitivity analysis. He calls this the process of structuring *a sensitivity analysis setting*. Saltelli (2002, 2008) and Saltelli and Tarantola (2002) identify main settings such as factor prioritization and factor fixing. Factor prioritization refers to identifying the inputs/parameters/variables on which to focus for reducing uncertainty or on which to draw managerial attention during the implementation phase. The methods used for factor prioritization range from techniques based on gradients and differentials (Iman & Helton, 1988; Tsanakas & Millossovich, 2016), to one‐at‐a‐time sensitivities displayed via tornado diagrams (Felli & Hazen, 2004; R. Howard, 1988; T. Reilly, 2000), to nonparametric statistical methods (Helton & Davis, 2002) to variance‐based methods (Iman & Hora, 1990), to moment‐independent methods (a comparison is offered in Borgonovo (2006)), to the value of information (Felli, 1998; Oakley, 2009). Factor fixing instead refers to finding the irrelevant model inputs with a limited number of model runs. A well‐known method for factor fixing is the method of Morris (1991), which is based on the randomized computation of one‐at‐a‐time sensitivities. Besides these two settings, the literature has identified interaction quantification and the determination of the direction of impact as two additional relevant settings. Some methods used for factor prioritization, such as tornado diagrams and gradients, provide insights into the direction of impact. Moreover, the literature recently proposed dedicated methods such as PD and ALE plots. We review traditional and more recent methods in the next section.

### 2.2 Direction of impact indicators

We consider an analyst who has available or has developed a quantitative risk assessment model to calculate or forecast one or more risk metrics of interest. We write the model as an input–output mapping which relates the quantity of interest Y to a set of input variables $X=[X_{1},X_{2},\text{…},X_{n}]$:

(1) Y=g(X)+E(X),

where g:X→R, $X\subseteq \;R^{n}$ denotes the underlying multivariate input–output mapping and E is a stochastic error term with zero mean, so that $E\left[Y|X=x^{0}\right]=g\left(x^{0}\right)$. We regard X, Y, and E(X) as random vectors on the reference space (Ω,B(Ω),P). Furthermore, let $F_{X}$ and $F_{Y}$ denote the cumulative distribution functions of the input and output, respectively, while $f_{X}$ and $f_{Y}$ denote their respective densities, if available. If E is absent or a constant random variable, the simulator is said to be deterministic. In the remainder, we set E=0, considering deterministic input–output mappings. For notation, it is useful to partition the domain X into $X_{i}\subseteq R$ and $X_{-i}\subseteq R^{n-1}$, with $X=X_{i}\times X_{-i}$, so that the notions x∈X, $x_{i}\in X_{i}$, $x_{-i}\in X_{-i}$, and $x=(x_{i};x_{-i})$ are defined. Hereafter, $X_{i}$ denotes an uncertain input, $x_{i}$ one of its realizations.

The selection of the ranges $X_{i}$ is central for a direction of impact analysis. Assigning too small ranges might result in missing significant insights on the output response. Even if probability distributions have not been formally designated, the ranges should cover the support of $X_{i}$, ensuring the model response is inspected over the entire set of values that $X_{i}$ can assume to the best of our knowledge. We present the indicators used in this work in Table 1.

Gradient‐based indicators (partial derivatives) are local indicators. Consider the Newton ratio

(2) $${\hat{g}}_{i}^{\prime }\left(x^{0}\right)=\frac{g_{i}\left(x_{i}^{0}+\Delta ,x_{-i}^{0}\right)-g_{i}^{\prime }\left(x^{0}\right)}{\Delta },$$

where $x^{0}$ and $(x_{i}^{0}+\Delta ;x_{-i})\in X$, Δ is an increment input $X_{i}$. Numerically, Equation (2) is an approximation of a partial derivative. The sign indicates whether the increase in $X_{i}$ from $x_{i}^{0}$ to $x_{i}^{0}+\Delta$ has a positive or negative impact on the model response. Second‐order Newton ratios can be used to obtain information about local concavity and convexity. Aside from the brute force definition in Equation (2), several methods have been developed to estimate gradients and Hessians efficiently, among which is automatic differentiation (Griewank, 2000).

Example 1 A classical toy model in risk assessment studies is the Ishigami function (Ishigami and Homma, 1990):

(3) $$y=g\left(x_{1},x_{2},x_{3}\right)=\sin\left(x_{1}\right)\left(1+0.1x_{3}^{3}\right)+7\sin{\left(x_{2}\right)}^{2}.$$

The inputs are traditionally assigned ranges $X_{i}=\left[-\pi ,\pi \right]$, so that $X={\left[-\pi ,\pi \right]}^{3}$. At $x^{0}=\left(0,0,0\right)$ we have the gradient vector $▽g\left(x^{0}\right)=\left[1,0,0\right]$, which indicates that the function is increasing in $x_{1}$ and stationary in $x_{2}$ and $x_{3}$.

**TABLE 1:** A selection of marginal effect indicators.

**Indicator**	**Symbol**	**Monotonicity** **consistency**	**Lipschitz** **consistency**	**Concavity** **consistency**	**Discrete** **inputs**	**Handles** **distributions**
Gradients/Hessians	$g_{i}^{\prime }\left(X\right)$	Yes	Yes	N/A	Yes	No
Tornado	$\Delta g_{i}^{+},\Delta g_{i}^{-}$	Yes	Yes	N/A	Yes	No
One‐way function	$g(x_{i};x_{-i}^{0})$	Yes	Yes	Yes	Yes	No
Correlation coefficient	$\rho_{Y,X_{i}}$	Independent	N/A	N/A	Yes	Yes
Conditional expectation	$r_{i}\left(x_{i}\right)$	Independent	No	Independent	Yes	Yes
Partial dependence function	${\text{PD}}_{i}\left(x_{i}\right)$	Yes	Yes	Yes	Yes	Yes
ALE function	${\text{ALE}}_{i}\left(x_{i}\right)$	Independent	No	Independent	Yes	Yes

A second popular indicator is represented by tornado diagrams (R. A, Howard, 1988). As discussed in T. Reilly (2000) and Felli and Hazen (2004), tornado diagrams can be built at different stages of the modeling process. In the early phases, we consider a reference value for the model inputs $x^{0}$ and assign corresponding ranges, without necessarily assessing probability distributions. Given $x^{0}$ and appropriate ranges $\left[x_{i}^{-},x_{i}^{+}\right]$ for each input, tornado diagrams evaluate the differences in the model response at the base case and at the extremes of the assigned ranges. The sensitivity measures are then the finite differences

(4) $$\begin{array}{ccc} \Delta_{i}^{+}g=g\left(x_{i}^{+},x_{-i}^{0}\right)-g\left(x^{0}\right) & \;\text{and}\; & \Delta_{i}^{-}g=g\left(x_{i}^{-},x_{-i}^{0}\right)-g\left(x^{0}\right). \end{array}$$

Note that, if input distributions were assigned with infinite support, $\left[x_{i}^{-},x_{i}^{+}\right]$ can then be quantiles (e.g., the 5% and 95% of these distributions).

**FIGURE 1:**
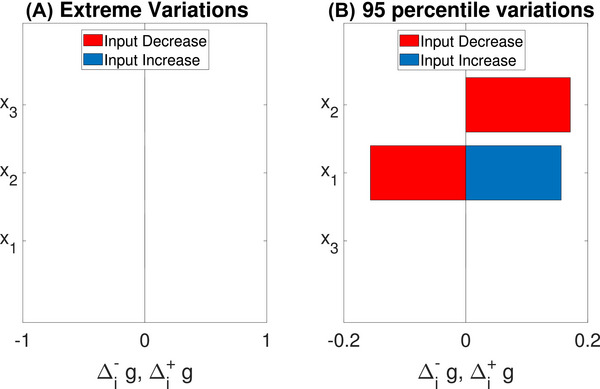
Two tornado diagrams for the Ishigami function.

Example 2 (Example 1 continued) For the Ishigami function and input variations on [−π,π], the tornado diagram appears empty (panel A in Figure 1). This occurs because the values at $g(x_{i}^{+},x_{-i}^{0})$ and $g(x_{i}^{-},x_{-i}^{0})$ are equal to $g(x^{0})$ for all inputs, so that $\Delta_{i}^{+}g=\Delta_{i}^{-}g=0$, i=1,2,3. We might then choose different extremes, for instance, selecting extremes that correspond to a hypothetical 90% confidence interval should the inputs be assigned uniform distributions. We then obtain the tornado diagram in panel B of Figure 1. This diagram now shows an effect for $X_{1}$, whose increase(decrease) causes the model output to increase(decrease). It also shows only the right bar for $X_{2}$, whose effect is, in this case, non‐monotonic. We find $\Delta_{i}^{+}g=\Delta_{i}^{-}g=0.17$, a positive effect both when $X_{2}$ increases and decreases. This shows a non‐monotonic dependence of the model on $X_{2}$.

The results in Example 2 show that we need to carefully interpret the indications of a tornado diagram. For instance, inferring from panel A that the Ishigami function does not depend on any input would be incorrect. Or, concluding that it does not depend on $X_{3}$ because panel B also does not display a sensitivity is also incorrect. The empty panel in panel A of Figure 1 is explained by the periodicity of the model and $\Delta g_{3}^{+}=0$ in panel B by the fact that fixing $X_{1}=0$ erases the dependence of Y on $X_{3}$.

A tornado diagram provides a snapshot of the model behavior across two scenarios in which we vary only one input and does not allow us to observe the intermediate behavior. We may then accompany such a representation with a one‐way sensitivity function that shows what happens for variations across the extremes. One‐way sensitivity functions have been extensively studied in decision and risk analysis (Bhattacharjya & Shachter, 2008; Castillo et al., 1997; Clemen, 1997; van der Gaag et al., 2007). They provide projections of the response surface in the direction of an input and are built as follows.

Fixed a baseline point $x^{0}\in X$, for each input we consider the function $g(x_{i};x_{-i}^{0})$ which fixes all coordinates but $x_{i}$ at their reference values ($x_{-i}^{0}$), while $x_{i}$ varies in $X_{i}$. Thus, for each input, there are as many one‐way sensitivity functions as there are baseline locations $x_{-i}^{0}$.

Example 3 (Example 1 continued) For the Ishigami function, using the ranges and base case as in Example 1, we have the one‐way sensitivity functions with respect to $X_{1}$, $X_{2}$, and $X_{3}$ reported in Table 2.

**TABLE 2:** Alternative marginal behavior indicators for the ishigami function.

$x^{0}=\left(0,0,0\right)$	$x_{1}$	$x_{2}$	$x_{3}$
$g_{i}^{\prime }\left(x^{0}\right)$	1	0	0
$g(x_{i};x_{-i}^{0})$	$\sin(x_{1})$	$7\sin{\left(x_{2}\right)}^{2}$	0
$r_{1}\left(x_{1}\right)={\text{PD}}_{i}\left(x_{i}\right)$	$2.95\sin(x_{1})+3.5$	$7\sin{\left(x_{2}\right)}^{2}$	3.5
${\text{ALE}}_{i}\left(x_{i}\right)$	$2.95\sin(x_{1})$	$7\sin{\left(x_{2}\right)}^{2}$	0

One‐way sensitivity functions can be visualized in separate graphs or, in a unique graph, via spiderplots. In a spiderplot, the origin represents the base case $\left(x^{0},g\left(x^{0}\right)\right)$. The horizontal axis reports the variation of the inputs on normalized ranges. This normalization allows the simultaneous representation of one‐way sensitivity functions in the same graph.

**FIGURE 2:**
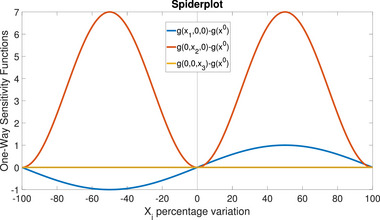
Ishigami model: A spiderplot with three one‐way sensitivity functions.

Example 4 (Example 1 continued) Figure 2 illustrates the spiderplot with the three one‐way sensitivity functions in Table 2. The vertical axis reports the values of $g\left(x_{i},x_{-i}^{0}\right)-g\left(x^{0}\right)$ for each input. The spiderplot in Figure 2 shows the sinusoidal behavior of the function with respect to $X_{1}$ and $X_{2}$. However, the graph of $g(0,0,x_{3})$ is flat. Fixing simultaneously $X_{1}=0$ and $X_{2}=0$ makes the function constant in $X_{3}$.

The indicators mentioned thus far provide local information and do not account for the type of distribution assigned to the inputs. A global approach requires us to perform the analysis giving full credit to the probability distributions. In risk assessment applications, one typically relies on Monte Carlo simulations to generate a dataset of input realizations that follow the assigned distributions. The risk assessment model is then run to obtain the corresponding values of the output. Then, a popular way to gain information about marginal behavior is to compute the correlation coefficients between Y and the inputs of interest. A positive (negative) sign of these coefficients indicates that g is expected to increase (decrease) with $X_{i}$.

Example 5 (Example 1 continued) For the Ishigami function, the inputs are assumed to be uniform random variables, independently distributed on [−π,π]. Uncertainty quantification under these input assumptions with uniform distributions yields a bimodal output distribution, with a mean equal to 3.5, and a variance of 13.85. Calculating the correlation coefficient between Y and $X_{i}$, we find $\rho_{Y,X_{1}}=0.437$, $\rho_{Y,X_{2}}=0$, and $\rho_{Y,X_{3}}=0$.

By the sign of the correlation coefficient, we would expect Y to be increasing in $X_{1}$, and we would obtain no insight into the impact of $X_{2}$ and $X_{3}$. Concluding that they do not play a role in the model would be an erroneous inference, as they do play a role in the model. The misleading indication from the correlation coefficient is well known: the correlation coefficient fails to capture the dependence between Y and $X_{i}$ when the input–output mapping is nonlinear.

Thus, analysts resort to marginal behavior indicators that do not assume linearity. *Conditional expectation functions* are a standard choice in statistics and econometrics (Wooldridge, 2013). They are defined as the conditional expectation of Y given $X_{i}$:

(5) $$r_{i}\left(x_{i}\right)=E\left[g\left(X\right)|X_{i}=x_{i}\right]=\int_{X_{-i}}g\left(x_{i};x_{-i}\right)dF_{X_{-i}|X_{i}}\left(x_{i};x_{-i}\right),$$

where $F_{X_{-i}|X_{i}}\left(x_{i};x_{-i}\right)$ is the conditional cumulative distribution function of $X_{-i}$ given $X_{i}$. The graphs of conditional expectation functions are part of data visualization tools such as conditioning plots (Chambers & Hastie, 1992). They are called M‐plots in Apley and Zhu (2020).

In the machine learning literature, Friedman (2001) introduces *PD functions* as marginal behavior indicators. These functions have been subsequently widely employed and studied (see Hooker (2007) and Goldstein et al. (2015)). Formally, a PD function is defined as

(6) $${\text{PD}}_{i}\left(x_{i}\right)=\int_{X_{-i}}g\left(x_{i};x_{-i}\right)dF_{X_{-i}}\left(x_{-i}\right),$$

A PD function communicates the behavior of g as a function of $X_{i}$, averaging over the marginal distribution of the remaining inputs. Equations (5) and (6) show that conditional expectation and PD functions are obtained by fixing $X_{i}=x_{i}$ and then averaging one‐way sensitivity functions $g(x_{i};x_{-i})$ over $X_{-i}$, but with different probability measures: the conditional measure $F_{X_{-i}|X_{i}}\left(x_{i};x_{-i}\right)$ in $r_{i}\left(x_{i}\right)$ and the marginal $F_{X_{-i}}\left(x_{-i}\right)$ in $PD_{i}\left(x_{i}\right)$. When inputs are independent these probability measures coincide and $r_{i}\left(x_{i}\right)=PD_{i}\left(x_{i}\right)$. When inputs are dependent, however, the two indicators provide different results. This difference has been analyzed from a numerical and data‐driven perspective in works such as Apley and Zhu (2020) and Hooker et al. (2021) (We refer to Chapter 8 of Molnar, 2018, for additional details.) The main implication is that relying on a conditional distribution avoids extrapolation issues, while using a marginal distribution may force the model to produce estimates on points far from the data where the model has been trained, with predictions becoming unreliable. In contrast, the mathematical implications of using a marginal rather than a conditional distribution have not yet been explored and will be the focus of the next section.

**FIGURE 3:**
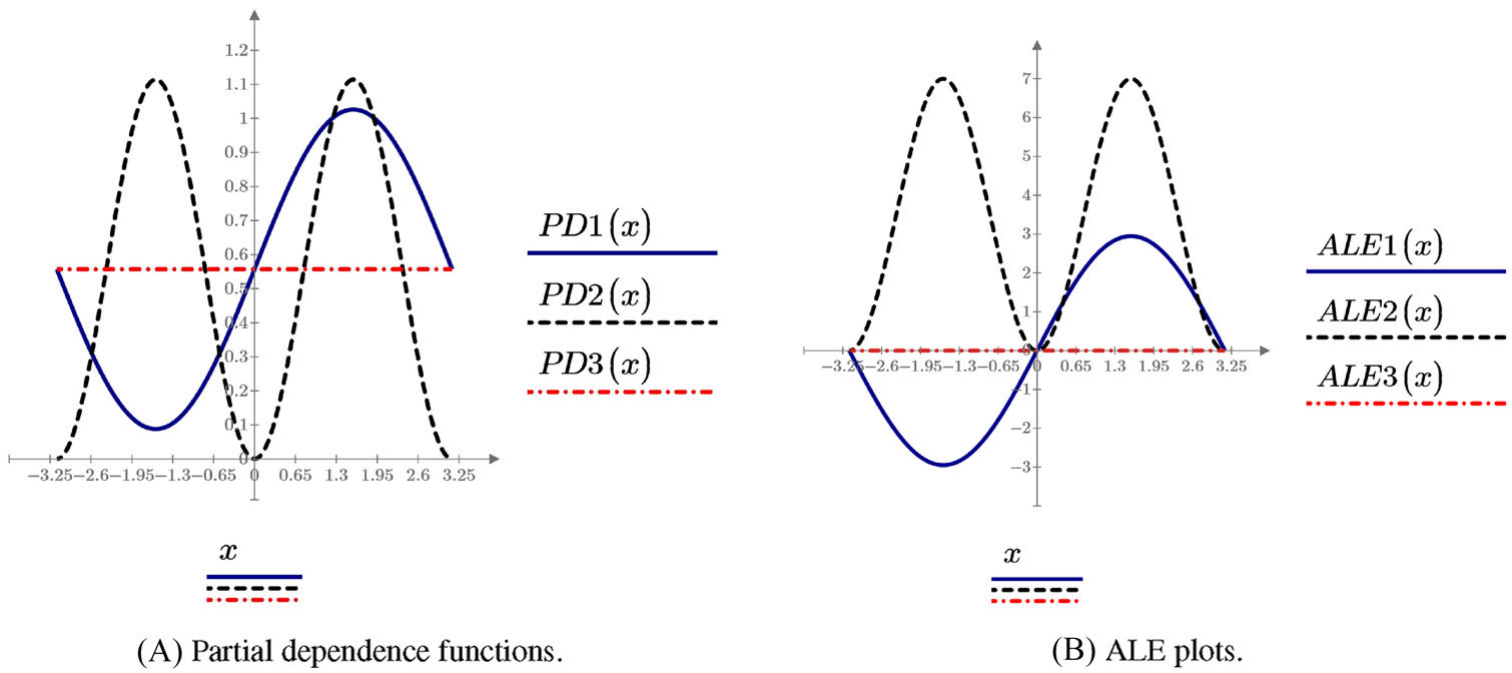
Ishigami model: PD (conditional expectation) functions, and ALE plots.

Example 6 (Example 1 continued) The PD (conditional expectation) functions for the Ishigami model are summarized in Table 2 and visualized in Figure 3A.

Figure 3A shows that the PD and conditional expectation plots correctly report the sinusoidal dependence of Y on $X_{1}$. One notes the difference between the PD function and the one‐way sensitivity function. Thus, there is a difference between the average dependence portrayed by a PD (conditional regression) function and the local dependence of a one‐way sensitivity function. The PD functions of $X_{2}$ exactly capture the sinusoidal dependence of Y on this input. This is a consequence of the additive recovery property, as discussed in Apley and Zhu (2020).

Finally, the PD plot of $X_{3}$, ${\text{PD}}_{3}\left(x_{3}\right)$, is flat. This result is a false negative due to what we can call the “flat expectation effect.” Specifically, let the input–output mapping be of the form $Y=g_{i}\left(x_{i}\right)g_{-i}\left(x_{-i}\right)$. If $g_{-i}\left(x_{-i}\right)$ is such that $\int_{X_{-i}}g_{-i}\left(x_{-i}\right)dF_{X_{-i}}\left(x_{-i}\right)=0$, then $PD_{i}\left(x_{i}\right)=0$.

Goldstein et al. (2015) propose to overlay PD plots with the graphs of multiple one‐way sensitivity functions in a graphical representation called an ICE plot.^1^ This representation allows analysts to visualize local information (one‐way sensitivity plots) and global information on marginal behavior (PD plots).

Recently, Hooker and Mentch (2019), Apley and Zhu (2020), and Molnar et al. (2024) highlight limitations of PD functions when inputs are correlated. The issue is that the numerical implementation of (6) may ask for forecasts in regions where no data are available. Extrapolation errors may result in misleading graphical representations of the input–output dependence yielding unreliable indications. To remedy this issue, Apley and Zhu (2020) propose ALE plots, defined as

(7) $${\text{ALE}}_{i}\left(x_{i}\right)=\int_{x_{i,\min}}^{x_{i}}E\left[g_{i}^{\prime }\left(X\right)|X_{i}=z_{i}\right]dz_{i},$$

where $g_{i}^{\prime }\left(X\right)$ is the partial derivative of g with respect to $X_{i}$ and $x_{i,\min}$ is the infimum of the support of $X_{i}$. This indicator is global as it requires the evaluation of partial derivatives at randomized locations of the input space, and the use of a conditional expectation reduces the extrapolation risk.

Example 7 (Example 1 continued) The ALE functions for the Ishigami model are reported in the last row of Table 2 and visualized in Figure 3B.

Figure 3B shows that the ALE plots exactly display the dependence of Y on $X_{2}$, similar to the PD and conditional expectation functions. In fact, as Apley and Zhu (2020) prove, they possess the additive recovery property. Similarly to PD functions, also the ALE plot of $X_{3}$ is flat, showing that also ALE plots are exposed to the flat expectation effect.

This review has presented the main indicators used in the literature to study marginal dependence. These indicators have been widely studied, especially from a numerical viewpoint. Our focus in the next section is on their theoretical properties.

## 3 WHICH INDICATOR IS CONSISTENT?

This section examines the behavior of marginal effect indicators with respect to three relevant properties that allow analysts to better understand the model response: monotonicity (Section 3.1), the Lipschitz property (Section 3.2), and concavity (Section 3.3).

### 3.1 Monotonicity

In studying monotonicity, an analyst is interested in understanding whether the model output increases or decreases as the input of interest varies over the assigned range.

Definition 1 (Monotonicity) A function g:X→R is separately increasing (decreasing) in $x_{i}$ if

(8) $$g\left(x_{i}+t_{i};x_{-i}\right)\overset{\left(\leq \right)}{\geq }g\left(x_{i};x_{-i}\right)$$

for all $t_{i}>0$, with $\left(x_{i}+t_{i};x_{-i}\right)$ and $(x_{i};x_{-i})\in X$.

We call a marginal behavior indicator *monotonicity consistent* if, given that g is separately monotonic with respect to $x_{i}$, the indicator reports such monotonicity. The rationale is self‐evident, for, if the analyst is aware that the simulator response is increasing in a certain input, she expects the graphical indicator to coherently communicate such behavior. A violation of consistency would either question the reliability of the indicator or require the analyst to carefully consider the interpretation of the graph.

We present results for the indicators in Table 1 starting with gradients. Definition 1 implies

(9) $${\hat{g}}_{i}^{\prime }\left(x\right)=\frac{g\left(x_{i}+t_{i};x_{-i}\right)-g\left(x_{i};x_{-i}\right)}{t_{i}}\overset{\left(\leq \right)}{\geq }0.$$

Thus, if g is increasing (decreasing) in $X_{i}$ and g has first‐order partial derivatives, their sign is consistently positive (negative). For tornado diagrams, the sensitivity measures $\Delta g_{i}^{+}\geq 0$ reflect the direction of the dependence of g on $X_{i}$. If g is increasing (decreasing) in $X_{i}$ on $X_{i}$, then $\Delta g_{i}^{+}\overset{\left(\leq \right)}{\geq }0$ when $X_{i}$ is shifted from $x_{i}^{0}$ to $x_{i}^{+}\left(x_{i}^{-}\right)$. Thus, the sensitivity measures of tornado diagrams are monotonicity consistent.

For one‐way sensitivity functions, setting $x_{i}=x_{i}^{0}+t_{i}$ in Definition 1 yields $g\left(x_{i};x_{-i}^{0}\right)\overset{\left(\leq \right)}{\geq }g\left(x^{0}\right)$ for any $\left(x_{i};x_{-i}^{0}\right)$ and $x^{0}\in X$. Therefore, one‐way sensitivity functions are monotonicity consistent.

Example 8 Let $g:X=R\times R_{\geq 0}\to R$, $\left(X_{1},X_{2}\right)\mapsto X_{1}X_{2}$. The mapping g is then increasing in $X_{1}$. Because $X_{2}$ is positive, one‐way sensitivity functions are increasing straight lines in $X_{1}$, for all $x_{2}^{0}$. We have $g\left(x_{1},x_{2}^{0}\right)=x_{1}x_{2}^{0}$. Similarly, the partial derivative $g_{1}^{\prime }\left(X_{1},X_{2}\right)=X_{2}$ is always positive.

Continuing with the indicators in Table 1, correlation coefficients and conditional expectation functions are not monotonicity consistent, as the next example shows.

Example 9 (Example 8 continued) Let $g(X_{1},X_{2})$ be as in Example 8, but with $X_{1}$ and $X_{2}$ having the joint probability distribution given in Table 3.

While $g(X_{1},X_{2})$ is separately increasing in $X_{1}$, we find a negative correlation coefficient $\rho_{Y,X_{1}}=-0.083$. The conditional expectation function $r_{1}\left(x_{1}\right)$ is not monotonic (Table 3, last column).

**TABLE 3:** Joint probability distribution $P(X_{1}=x_{1},X_{2}=x_{2})$ for Example 9.

	$X_{1}=1$	$X_{1}=2$	$X_{1}=3$	$P(X_{2}=x_{i})$	$r_{1}\left(x_{1}\right)$
$X_{2}=1$	0.25	0.10	0.01	0.36	2.06
$X_{2}=2$	0.30	0.01	0.01	0.32	1.25
$X_{2}=3$	0.30	0.01	0.01	0.32	2.00
$P(X_{1}=x_{i})$	0.85	0.12	0.03	1	

However, we can find a sufficient condition under which conditional expectation functions become monotonicity consistent (see Appendix A for all proofs).

Proposition 1 (Conditional expectation functions: monotonicity) A sufficient condition for the conditional expectation function $r_{i}\left(x_{i}\right)$ to be monotonicity consistent is that

(10) $$\begin{array}{c} f_{X_{-i}}\left(X_{-i}|X_{i}=x_{i}+\Delta \right)g\left(x_{i}+\Delta ;X_{-i}\right)\overset{\left(\leq \right)}{\geq }\\ f_{X_{-i}}\left(X_{-i}|X_{i}=x_{i}\right)g\left(x_{i};X_{-i}\right) \end{array}$$

holds for all $\left(x_{i}+\Delta ;X_{-i}\right)$, $(x_{i};X_{-i})\in X$.

Condition (10) is restrictive because it limits variations in g and the conditional input density tying them. Controlling these quantities is impossible in applications. However, if $X_{-i}$ is independent of $X_{i}$, Condition (10) is satisfied, because $f_{X_{-i}}\left(X_{-i}|X_{i}=x_{i}+\Delta \right)=f_{X_{-i}}\left(X_{-i}|X_{i}=x_{i}\right)$ and we are left with $g\left(x_{i}+\Delta ;X_{-i}\right)\geq f_{X_{-i}}\left(X_{-i}|X_{i}=x_{i}\right)g\left(x_{i};X_{-i}\right)$, which is true if g is increasing. Thus, it is the statistical dependence between $X_{i}$ and $X_{-i}$ that makes conditional expectation functions lose consistency. This result generalizes the findings in Beccacece and Borgonovo (2011) where independence of all inputs was assumed to show that $r_{i}\left(x_{i}\right)$ is monotonicity consistent.

We now turn to PD functions.

Proposition 2 (PD functions: monotonicity consistency) Consider $\left(X,B\left(X\right),P_{X}\right)$, with $X=X_{1}\times X_{2}\times \cdots \times X_{d}$. If g is separately increasing (decreasing) in $X_{i}$, then ${\text{PD}}_{i}\left(x_{i}\right)$ is increasing (decreasing).

Proposition 2 does not assume independence. Thus, PD functions are monotonicity consistent even when inputs are dependent. However, the fact that a partial dependence function ${\text{PD}}_{i}\left(x_{i}\right)$ is increasing (decreasing) is not sufficient to guarantee that the input–output mapping is monotonic. Some sufficient conditions are possible when the input–output mapping g is additively separable or multiplicatively separable.

We say that a mapping g is additively separable in $X_{i}$ if it takes the form

(11) $$g\left(x\right)=a_{i}\left(x_{i}\right)+A_{-i}\left(x_{-i}\right),$$

where $a_{i}:X_{i}\to R$, and $A_{-i}:X_{-i}\to R$. We say that g is multiplicatively separable in $X_{i}$ if it can be written as

(12) $$g\left(x\right)=m_{i}\left(x_{i}\right)M_{-i}\left(x_{-i}\right).$$

Apley and Zhu (2020, p. 6) observe that PD functions respect these factorizations and state that they possess an *additive* and a *multiplicative recovery* property. We next link these properties to monotonicity in the next corollary.

Corollary 1 (PD functions: separability and monotonicity) (1) Let g be additively separable in $X_{i}$ (Equation 11). If ${\text{PD}}_{i}\left(x_{i}\right)$ is increasing (decreasing) in $x_{i}$, then g is increasing (decreasing) in $x_{i}$.

(2) Let g be multiplicatively separable in $X_{i}$ (Equation 12) and $M_{i}=\int M_{-i}\left(x_{-i}\right)dF_{-i}\left(x_{-i}\right)\neq 0$. Then, if $M_{i}>0\left(M_{i}<0\right)$, ${\text{PD}}_{i}\left(x_{i}\right)$ increasing in $x_{i}$ implies g increasing (decreasing) in $x_{i}$.

Combined with Proposition 2, the first item in Corollary 1 suggests that, for an additively separable g, ${\text{PD}}_{i}\left(x_{i}\right)$ is increasing (decreasing) in $X_{i}$ if and only if g is increasing (decreasing).

We come to ALE functions. In general, it is not possible to prove that ALE functions are monotonicity consistent. However, to better interpret them, let us recall how they are computed. First, we take the conditional expectation of derivatives (or numerically Newton ratios) and then we accumulate them. The sign of partial derivatives is consistent with the monotonicity of g. Thus, if g is increasing, then ALE functions will be positive, as we are accumulating positive terms.

Example 10 (Example 8 continued) Consider the same input–output mapping as in Example 8, now with $X_{1}$ and $X_{2}$ jointly exponentially distributed with the Gumbel (1960) cumulative distribution function $F_{X}\left(x_{1},x_{2};\delta \right)=1-e^{-x_{1}}-e^{-x_{2}}+e^{-(x_{1}+x_{2}+\delta x_{1}x_{2})}$, 0≤δ≤1. The function ${\text{ALE}}_{1}\left(x_{1}\right)$ can be computed analytically:

(13) $${\text{ALE}}_{1}\left(x_{1}\right)=\left\{\begin{array}{cc} \frac{\left(\delta x_{1}+1\right)\ln\left(\delta x_{1}+1\right)+\delta^{2}x_{1}}{\delta^{2}x_{1}+\delta } & \text{if}\;\delta \neq 0,\\ x_{1} & \text{if}\;\delta =0. \end{array}\right.$$

**FIGURE 4:**
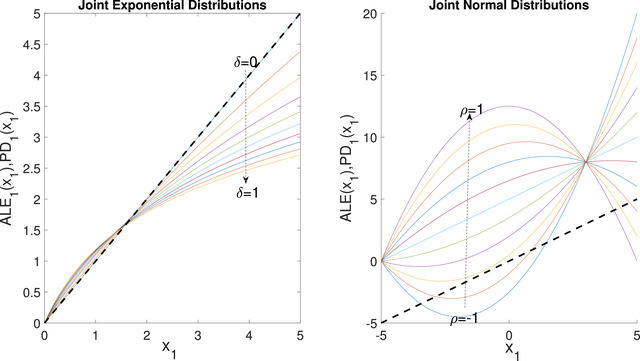
${\text{ALE}}_{1}\left(x_{1}\right)$ (solid lines) and ${\text{PD}}_{1}\left(x_{1}\right)$ (dashed line) for the product model with the distributions in Examples 10 (left panel) and 11 (right panel).

Figure 4 displays the graphs of ${\text{ALE}}_{1}\left(x_{1}\right)$ for different values of δ. If δ=0, then $\;{\text{ALE}}_{1}\left(x_{1}\right)={\text{PD}}_{1}\left(x_{1}\right)=x_{1}$ (dotted line). If δ>0 then ${\text{ALE}}_{1}\left(x_{1}\right)$ is positive, consistent with the fact that g is increasing in $X_{1}$. However, the graph of ${\text{ALE}}_{1}\left(x_{1}\right)$ displays a nonlinear dependence. Conversely, ${\text{PD}}_{1}\left(x_{1}\right)\;=\;x_{1}$ is independent of δ.

The right panel in Figure 4 shows that for δ>0 the ALE functions present a curvature which is not present in the original marginal dependence of Y on $X_{1}$. Formally, as shown in Apley and Zhu (2020), the function ${\text{ALE}}_{i}\left(x_{i}\right)$ does not possess the multiplicative recovery property when inputs are dependent. An exception is represented by the case of additively separable mappings for which ALE plots are monotonicity consistent thanks to the additive‐recovery property.

Proposition 3 (ALE functions: monotonicity consistency for additive models) Consider any $\left(X,B\left(X\right),P_{X}\right)$. If g is separately monotonic in $X_{i}$, then the function ${\text{ALE}}_{i}\left(x_{i}\right)$ is monotonicity consistent in sign.

The next example discusses a different case in which the input support is the real line.

Example 11 (Example 10 continued) Consider the input–output mapping $Y=X_{1}X_{2}$ as in Example 8. However, let now $X_{1}$ and $X_{2}$ be jointly normal with correlation coefficient ρ and mean value and variance equal to unity. The function ${\text{ALE}}_{1}\left(x_{1}\right)$ in this case can be written as (see Appendix D):

(14) $${\text{ALE}}_{1}\left(x_{1}\right)=\left(1-\rho \right)\left(x_{1}-x_{min,1}\right)-\frac{\rho }{2}\left(x_{1}^{2}-x_{min,1}^{2}\right).$$

Note that if one sets $x_{min,1}$ at −∞, ${\text{ALE}}_{1}\left(x_{1}\right)$ is ill‐posed for any value of ρ, and otherwise ${\text{ALE}}_{1}\left(x_{1}\right)$ depends on an arbitrary $x_{min,1}$; this is a potential shortcoming of ALE plots when the support of $X_{1}$ is unbounded. In the graph of the right panel of Figure 4, we set $x_{min,1}=-5$. The linear behavior is recovered only for ρ=0.

### 3.2 Hölder and Lipschitz continuity

Besides knowing whether g is increasing or decreasing in $X_{i}$, an analyst may wish to know whether this growth is bounded. The related mathematical properties are Lipschitz‐continuity and α‐Hö lder continuity, from which we start.

We say that a multivariate function g satisfies the Hölder condition of order α, α>0, if it is true that

(15) $$|g\left(x^{0}\right)-g\left(x^{1}\right)|\leq H_{\alpha }{\left\|x^{0}-x^{1}\right\|}^{\alpha }$$

with $H_{\alpha }>0$, for all for all $x^{0}$ and $x^{1}$ in X. We note that if a function satisfies the Hölder condition with α=1, then it is Lipschitz continuous and we write

(16) $$|g\left(x^{0}\right)-g\left(x^{1}\right)|\leq L\left\|x^{0}-x^{1}\right\|,$$

where $L\in R_{+}$ is the Lipschitz constant. In the remainder, we shall use the abbreviation α‐Hölder or “Lipschitz” instead of “Lipschitz (H ölder) continuous”. We say that g is separately H ölder in $x_{i}$ if

(17) $$|g\left(x_{i}^{1};x_{-i}^{0}\right)-g\left(x_{i}^{0};x_{-i}^{0}\right)|\leq H_{\alpha }{\left|x_{i}^{1}-x_{i}^{0}\right|}^{\alpha },$$

for all $\left(x_{i}^{1};x_{-i}^{0}\right)$ and $(x_{i}^{0};x_{-i}^{0})\in X$. It is easy to see that, if g is α‐H ölder, then it is also separately α‐Hölder in any $X_{i}$. Separately Lipschitz refers to the case α=1 in (17).

We start with tornado diagrams. Considering the variation from $x_{i}^{0}$ to $x_{i}^{+}$, by Equations (4) and (17), we have

(18) $$|g\left(x_{i}^{+};x_{-i}^{0}\right)-g\left(x^{0}\right)\left|=\right|\Delta_{i}^{+}g|\leq H_{\alpha }{\left|x_{i}^{+}-x_{i}^{0}\right|}^{\alpha }.$$

Thus, if g is α‐Hölder, the sensitivity measures of tornado diagrams are bounded. The same holds for $\Delta_{i}^{-}g$. Regarding one‐way sensitivity functions, g is separately α‐Hö lder in $X_{i}$, if and only if $g(x_{i};x_{-i}^{0})$ is α‐H ölder in $X_{i}$ for all $x_{-i}^{0}$. Therefore, if one or more one‐way sensitivity functions are not α‐Hölder, g is not.

We then continue with PD functions. The following holds.

Proposition 4 (PD functions: Hölder consistency) If g is separately α‐Hölder in $X_{i}$, then ${\text{PD}}_{i}\left(x_{i}\right)$ is α‐Hölder with the same constant $H_{\alpha }$.

An immediate consequence is that if g is separately Lipschitz in $X_{i}$, then ${\text{PD}}_{i}\left(x_{i}\right)$ is Lipschitz with the same constant L.

We then consider conditional expectation functions $r_{i}\left(x_{i}\right)$. We know that they provide equivalent information as PD functions under input independence. However, there is no link between conditional expectation and α‐Hölder continuity under input dependence. The reason is technical, as discussed in Appendix B.

We finally come to ALE functions. The following holds.

Proposition 5 (ALE functions and Lipschitz continuity) If g is separately Lipschitz in $X_{i}$ and differentiable on X, then

(19) $$\begin{array}{c} \left|{\text{ALE}}_{i}\left(x_{i}^{1}\right)-{\text{ALE}}_{i}\left(x_{i}^{0}\right)\right|\leq L\left(T_{i}\left(x_{i}^{1}\right)-T_{i}\left(x_{i,\min}\right)\right.\\ +\left.T_{i}\left(x_{i}^{0}\right)-T_{i}\left(x_{i,\min}\right)\right), \end{array}$$

where $T_{i}\left(x_{i}\right)$ is the antiderivative of $t_{i}\left(x_{i}\right)=\int_{X_{-i}}f_{X_{i}|X_{-i}}\left(x_{i},x_{-i}\right)dx_{-i}$.

Thus, ALE plots retrieve a Lipschitz‐type inequality, but boundedness is governed by the function $T_{i}\left(x_{i}\right)$, rather than by the distance between $x_{i}^{1}$ and $x_{i}^{0}$, which is at the heart of the Lipschitz property. This reveals a weaker link between the Lipschitz property and ${\text{ALE}}_{i}\left(x_{i}\right)$ plots: Even if ${\text{ALE}}_{i}\left(x_{i}\right)$ is not Lipschitz, g(x) may be (separately) Lipschitz in $X_{i}$.

Example 12 Consider the following input–output mapping $g\left(x\right)=x_{1}+x_{2}$, with $X={\left[0,1\right]}^{2}$. Then, let $X_{1}$ be uniformly distributed on [0,1] and $X_{2}$ distributed with the following conditional density:

(20) $$f_{X_{2}|X_{1}}\left(x_{1},x_{2}\right)=\left\{\begin{array}{cc} \frac{1}{2}x_{2}^{-\frac{1}{2}} & \text{if}\;\frac{1}{2}\leq x_{1}\leq 1\\ 1 & \text{if}\;0\leq x_{1}<\frac{1}{2} \end{array}\right..$$

For the partial dependence function ${\text{PD}}_{2}\left(x_{2}\right)$, we find

(21) $${\text{PD}}_{2}\left(x_{2}\right)=\int_{0}^{1}\left(x_{1}+x_{2}\right)dx_{1}=x_{2}+\frac{1}{2},\;$$

which is Lipschitz on [0,1]. For the ALE function, we find

(22) $${\text{ALE}}_{2}\left(x_{2}\right)=\int_{0}^{x_{2}}\left(\frac{1}{2}+\frac{1}{4z^{1/2}}\right)dz=\left(\frac{1}{2}x_{2}+\frac{1}{2}\sqrt{x_{2}}\right),\;$$

which is not Lipschitz on [0,1].

### 3.3 Concavity

In this section, we aim at addressing whether the concavity/convexity of the model response is preserved by the marginal effect indicators studied in the previous sections. For presentation simplicity, we focus on concavity, but similar results hold for convexity.

Consider g:X→R, with X now a concave subset of $R^{n}$, which is the Cartesian product $X=X_{1}\times X_{2}\times \cdots \times X_{n}$ of concave univariate subsets for all i=1,2,⋯,n. We start with recalling that a function is concave if

(23) $$\begin{array}{c} g\left(\theta x^{1}+\left(1-\theta \right)x^{0}\right)\geq \theta g\left(x^{1}\right)+\left(1-\theta \right)g\left(x^{0}\right),\;\\ \text{for all}\;\;x^{1},x^{0}\in X,\;0\leq \theta \leq 1, \end{array}$$

and *strictly concave* if

(24) $$\begin{array}{c} g\left(\theta x^{1}+\left(1-\theta \right)x^{0}\right)>\theta g\left(x^{1}\right)+\left(1-\theta \right)g\left(x^{0}\right),\;\\ \text{for all}\;\;x^{1},x^{0}\in X,\;x\neq x^{0},\;0<\theta <1. \end{array}$$

To connect these concavity properties with marginal effect indicators, we start with one‐way sensitivity functions.

We start with local differential indicators, Hessians, and one‐way sensitivity functions. Hessians indicate concavity at a point $x^{0}$, with the second‐order test suggesting that if the input–output mapping is twice differentiable with continuous derivatives and convex at $x^{0}$, then the Hessian matrix is positive definite. If this occurs at all points in X, then the model is convex on X.

For a one‐way sensitivity function, we have the following.

Proposition 6 (One‐way sensitivity and concavity) One‐way sensitivity functions are concavity consistent.

Continuing with the indicators in Table 1, correlation coefficients are not designed to detect convexity. Conditional expectation functions are not concavity consistent when inputs are correlated.

For PD functions, the following holds.

Proposition 7 (PD functions: concavity consistency) If g is concave on X then ${\text{PD}}_{i}\left(x_{i}\right)$ is concave on $X_{i}$.

Similarly to the case of monotonicity, the concavity of all PD functions does not imply the overall concavity of g. Some sufficient conditions are reported below.

Corollary 2 (PD functions: concavity and separability) Under the assumptions of Corollary 1, (1) If g is additively separable in $x_{i}$, then it is concave in $x_{i}$ if and only if ${\text{PD}}_{i}\left(x_{i}\right)$ is concave in $x_{i}$. (2) If g is multiplicatively separable in $x_{i}$ and $\int M_{-i}\left(x_{-i}\right)dF_{-i}\left(x_{-i}\right)>0$ then ${\text{PD}}_{i}\left(x_{i}\right)$ is concave in $x_{i}$ if and only if g is concave in $x_{i}$. If $\int M_{-i}\left(x_{-i}\right)dF_{-i}\left(x_{-i}\right)<0$, then ${\text{PD}}_{i}\left(x_{i}\right)$ is convex if and only if g is concave in $x_{i}$.

We recall that a function is called additively concave on (convex domain) X if it can be written as $g=\sum_{i=1}^{n}g_{i}\left(x_{i}\right)$, with $g_{i}\left(x_{i}\right)$ univariate concave functions on (convex) $X_{i}$ (see Gong & Wang, 2021, for a review and applications of this notion). Then, Corollary 2 implies that g is additively concave if and only if ${\text{PD}}_{i}\left(x_{i}\right)$ is concave for all i=1,2,⋯,n — and ${\text{PD}}_{i}\left(x_{i}\right)$ differs from g only by an additive constant.

Finally, under input independence, the concavity consistency of PD functions implies the consistency of conditional expectation functions, as the two indicators coincide in this case.

Univariate first‐order ALE functions do not necessarily capture the concavity of an input–output mapping. In fact, they are based on first‐order differential information, which does not capture concavity by construction. However, the additive recovery property guarantees that when g is additive in $X_{i}$, and $g_{i}\left(x_{i}\right)$ is concave, then the first‐order ALE function is concave because ${\text{ALE}}_{i}\left(x_{i}\right)$ equals $g_{i}\left(x_{i}\right)$. This result holds also for multiplicative functions under input independence. In an ALE framework, concavity information is provided by second‐order ALE functions:

(25) $${\text{ALE}}_{i,j}\left(x_{i},x_{j}\right)=\int_{x_{\min,i}}^{x_{i}}\int_{x_{\;\min,j}}^{x_{j}}E\left[g_{i,j}^{\prime \prime }\left(X\right)|X_{i}=t,X_{j}=z\right]dtdz.$$

We refer to Apley and Zhu (2020) for additional details on second‐order ALE plots.

## 4 AFFINE MULTILINEAR MODELS

A clear understanding of what drives the indication of a graphical tool is generally not possible because closed‐form expressions are often out of reach. An exception is multilinear input–output mappings, which we now examine. Multilinear responses appear frequently in risk analysis, as this is the input–output mapping form in the case of Bayesian networks (Darwiche, 2003; Bhattacharjya & Shachter, 2010, 2012), reliability polynomials (Borgonovo & Smith, 2011), certain families of multicriteria utility functions (Bordley & Kirkwood, 2004) and, in simulation, multilinear response surfaces (Kleijnen, 2017).

Let Z={1,2,⋯,n} be the set of all model input indices and $Z=2^{Z}$ the corresponding power set. An affine multilinear function takes the form

(26) $$g^{ML}\left(x\right)=\sum_{z\in Z}\beta_{z}\prod_{s\in z}x_{s},$$

with the convention that the sum and product on the empty set yield 0 and 1, respectively.

Proposition 8 Given $\left(X,B\left(X\right),P_{X}\right)$, with $X=X_{1}\times X_{2}\times \cdots \times X_{n}$, let $g^{ML}:X\to R$. Denote the standard deviations of $X_{i}$ and $g^{ML}\left(X\right)$ by $\sigma_{i}=\sqrt{V[X_{i}]}$ and $\sigma_{Y}=\sqrt{V[g^{ML}\left(X\right)]}$. Then,

1. For partial derivatives, we have $g_{i}^{\prime }\left(x\right)=a_{-i}\left(x_{-i}\right)$,
2. Any one‐way sensitivity function is linear and written as
(27) $$g^{ML}\left(x_{i};x_{-i}\right)=a_{-i}\left(x_{-i}\right)x_{i}+b_{-i}\left(x_{-i}\right),$$
  where, $a_{-i}\left(x_{-i}\right)=\sum_{z\in Z:i\in z}\beta_{z}\prod_{s\in z,s\neq i}x_{s}$ and $b_{-i}\left(x_{-i}\right)=\sum_{z\in Z:i\notin z}\beta_{z}\prod_{s\in z}x_{s}$.
3. Correlation coefficients, conditional expectation, PD, and first‐order ALE functions are given, respectively, by
(28) $$\begin{array}{c} \begin{array}{cc} \rho_{Y\;X_{i}} & =\frac{1}{\sigma_{Y}\sigma_{i}}\left(E\left[a_{-i}\left(X_{-i}\right)X_{i}^{2}+b_{-i}\left(X_{-i}\right)X_{i}\right]\right.\\ & \;\left.-{\bar{x}}_{i}E\left[a_{-i}\left(X_{-i}\right)X_{i}+b_{-i}\left(X_{-i}\right)\right]\right), \end{array} \end{array}$$
(29) $$\begin{array}{c} r_{i}\left(x_{i}\right)=x_{i}E\left[a_{-i}\left(X_{-i}\right)|X_{i}=x_{i}\right]+E\left[b_{-i}\left(X_{-i}\right)|X_{i}=x_{i}\right], \end{array}$$
(30) $$\begin{array}{c} {\text{PD}}_{i}\left(x_{i}\right)=x_{i}{\bar{a}}_{-i}+{\bar{b}}_{-i},\;\text{and} \end{array}$$
(31) $$\begin{array}{c} {\text{ALE}}_{i}\left(x_{i}\right)=\int_{x_{\min,i}}^{x_{i}}E\left[a_{-i}\left(X_{-i}\right)|X_{i}=t\right]dt, \end{array}$$
  where ${\bar{a}}_{-i}=E\left[a_{-i}\left(X_{-i}\right)\right]$, ${\bar{b}}_{-i}=E\left[b_{-i}\left(X_{-i}\right)\right]$ and ${\bar{x}}_{i}=E\left[X_{i}\right]$.
4. If $X_{i}$ is independent of $X_{-i}$, then
(32) $$\begin{array}{cc} \rho_{Y\;X_{i}} & =\frac{{\bar{a}}_{i}\sigma_{i}}{\sigma_{Y}}, \end{array}$$
(33) $$\begin{array}{cc} r_{i}\left(x_{i}\right) & ={\text{PD}}_{i}\left(x_{i}\right)={\bar{a}}_{-i}x_{i}+{\bar{b}}_{-i},\;\text{and} \end{array}$$
(34) $$\begin{array}{cc} {\text{ALE}}_{i}\left(x_{i}\right) & ={\bar{a}}_{-i}\left(x_{i}-x_{\min,i}\right). \end{array}$$

Equations (27) and (30) reveal that one‐way‐sensitivity and PD functions are linear in $X_{i}$, independent of whether the inputs are dependent. The slope of a one‐way sensitivity function, $a_{-i}\left(x_{-i}^{0}\right)$, is the local slope of the multilinear map at $x^{0}$. In contrast, the slope of a PD function, ${\bar{a}}_{-i}$, is the average slope of the input–output mapping. Specifically, ${\bar{a}}_{-i}$ is equal to the mean value of the partial derivative of $g^{ML}\left(X\right)$ with respect to $X_{i}$. Thus, PD functions deliver average information about the marginal behavior of Y as a function of $X_{i}$.

Regarding conditional expectation functions, the linear term $x_{i}$ in Equation (29) is multiplied by the conditional expectation of the slope $a_{-i}\left(X_{-i}\right)$, which is a function of $X_{i}$. Similarly, the conditional expectation of the intercept $E[b_{-i}\left(X_{-i}\right)|X_{i}=x_{i}]$ depends on $X_{i}$. Hence, the inconsistency of $r_{i}\left(x_{i}\right)$ is generated by the dependence of the conditional expectation on $X_{i}$. In contrast, the marginal expectation associated with PD functions makes the values of the slope $E_{X_{-i}}\left[a_{-i}\left(X_{-i}\right)\right]$ and of the intercept $E_{X_{-i}}\left[b_{-i}\left(X_{-i}\right)\right]$ independent of $X_{i}$, thus preserving the linear dependence (see Example 13).

Regarding individual ALE functions, Equations (31) and (34) show that these functions are linear in the independent input case but may not be linear in the dependent case. Similarly to the case of conditional expectation functions, the potential nonlinearity is induced by the dependence of the conditional expectation on $X_{i}$. Regarding correlation coefficients, Equation (32) shows that when inputs are independent $\rho_{Y,X_{i}}$ is proportional to the slope of ${\text{PD}}_{i}\left(x_{i}\right)$, hence also consistent with PD functions. This is not true if inputs are dependent.

Example 13 Consider the multilinear input‐output mapping $g^{ML}\left(X_{1},X_{2},X_{3}\right)=X_{1}X_{2}+X_{1}X_{3}$ with X multivariate normal with mean μ=[1,1,1] and variance–covariance matrix Σ equal to the identity. Under independence, we have that $\rho_{Y,X_{i}}=\begin{array}{ccc} {}[0.70, & 0.35, & 0.35]. \end{array}$ The PD functions are

(35) $$\begin{array}{cccccc} {\text{PD}}_{1}\left(x_{1}\right) & =2x_{1},\;\; & {\text{PD}}_{2}\left(x_{2}\right) & =x_{2}+1,\;\; & {\text{PD}}_{3}\left(x_{3}\right) & =x_{3}+1. \end{array}$$

We also have $r_{i}\left(x_{i}\right)={\text{PD}}_{i}\left(x_{i}\right)$ because inputs are independent. Note that in this example the input‐output mapping is symmetric in $X_{2}$ and $X_{3}$ and so are the indicators $\rho_{YX_{2}}=\rho_{YX_{3}}$, and ${\text{PD}}_{2}\left(s\right)={\text{PD}}_{3}\left(s\right)$. Suppose that new data lead to a reassessment of the input distributions imposing a negative correlation $\rho_{1,2}=-0.75$ between $X_{1}$ and $X_{2}$, while the other input pairs remain independent. The correlation coefficients become $\rho_{YX_{i}}=\left[0.53,-0.212,0.424\right]$. Thus, $\rho_{Y\;X_{2}}$ is no longer equal to $\rho_{Y\;X_{3}}$ and differs from $\rho_{Y\;X_{1}}$ in sign.

**FIGURE 5:**
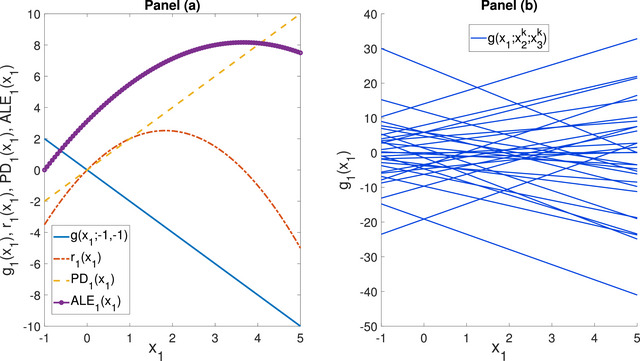
Panel (A: Alternative marginal behavior indicators for Example 13 in the dependent input case. Panel B: One‐way sensitivity functions at k=1,2,⋯,30 different baseline points $\left(x_{2}^{k},x_{3}^{k}\right)$.

Figure 5 reports the graphs of $g(x_{1};-1,-1)$, $r_{1}\left(x_{1}\right)$, ${\text{PD}}_{1}\left(x_{1}\right)$ and ${\text{ALE}}_{1}\left(x_{1}\right)$ in the dependent input case. We observe that $r_{1}\left(x_{1}\right)$ is nonlinear, which is due to the exponential dependence brought up by the normal density function.^2^ Similarly, ${\text{ALE}}_{1}\left(x_{1}\right)$ is nonlinear. Conversely, ${\text{PD}}_{1}\left(x_{1}\right)$ and $g(x_{i};-1;-1)$ are linear, consistently with the multilinear dependence of g on $X_{1}$. The fact that $g(x_{1};-1;-1)$ is decreasing while ${\text{PD}}_{1}\left(x_{1}\right)$ is increasing and that ${\text{ALE}}_{1}\left(x_{1}\right)$ changes sign indicates that the input–output mapping is non‐monotonic. The one‐way sensitivity functions in the ICE plot of panel B confirm this insight.

These observations and results extend naturally to the case of multilinear models.

## 5 INTERVENTION VERSUS STATISTICAL ASSOCIATION

The marginal behavior analysis can help risk analysts in planning an intervention or for information (forecasting) purposes. For an intervention, an analyst asks a question of the type: on which variable should we act to decrease the value of the risk metric? For forecasting, the analyst is interested in predicting the risk metric after she receives new information about one or more of the variables. Consistency is relevant in both contexts because it guarantees that the marginal behavior indicator correctly reflects the response of the risk metric.

**FIGURE 6:**
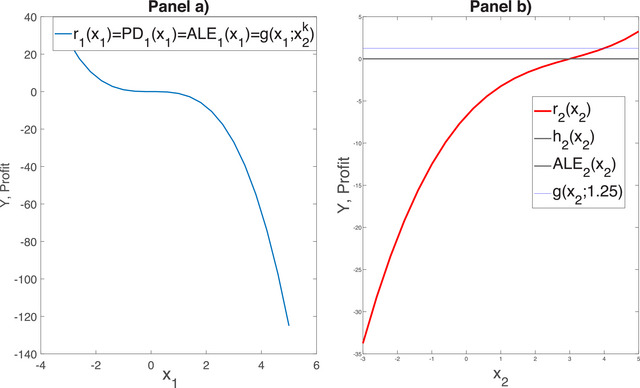
Marginal behavior analysis for the input–output mapping in Example 14.

Example 14 In a given risk assessment problem, the risk metric depends on two inputs through $Y=g\left(X\right)=-X_{1}^{3}$, with $X_{1}$ and $X_{2}$ jointly normal, with $\mu_{1}=\mu_{2}=\sigma_{1}=\sigma_{2}=1$ and $\rho_{1,2}=-0.5$. The analyst is asked to assess whether it is possible to act on $X_{2}$ to decrease the value of the risk metric, because acting on $X_{1}$ is too expensive. Suppose that analysts decide to use a conditional expectation function to display the behavior of Y as a function of $X_{1}$ and $X_{2}$, obtaining the graph reported in panel B of Figure 6.

By the conditional expectation function $r_{i}\left(x_{i}\right)$, the analyst would recommend to decrease $X_{2}$ because, in expectation, such a decrease leads to a decrease in Y (this is the interpretation of the graph of $r_{2}\left(x_{2}\right)$). However, because the risk metric depends functionally only on $X_{1}$ and $X_{1}$ is not subject to change, reducing $X_{2}$ will not result in any decrease in the value of the risk metric. This is informed by the ICE,^3^ PD, or ALE plots in panel B, which are flat lines. Note the difference with respect to an information perspective, where we are informed that $X_{2}$ is going to decrease tomorrow. In that case, because we know that $X_{2}$ is negatively correlated with $X_{1}$, we would expect that $X_{1}$ would increase. Then, panel A in Figure 6 shows that all indicators inform us that the risk is decreasing if $X_{1}$ increases. Thus, we can expect a decrease in the value of the risk metric tomorrow if we are informed that $X_{2}$ decreases.

## 6 APPLICATIONS

This section is divided into two parts. In Section 6.1, we present insights gained by the application of PD‐ICE plots to probabilistic risk assessment models. In Section 6.2, we present a case study on a susceptible exposed infected recovered (SEIR) model developed during the COVID‐19 pandemic.

### 6.1 Probabilistic safety assessment modeling

Starting with the foundational work of Kaplan and Garrick (1981), alternative formulations of the notation of risk have been developed. Aven (2012) and Aven (2018) offer, respectively, a historical overview and a broad perspective on the definition of risk within the risk science discipline. Space limitations force us to provide a cursory review, aimed at evidencing the mathematical aspects. Kaplan and Garrick express the risk associated with a given system or human enterprise as the triplet R={S,P,C}, where S is the set of all possible scenarios, P is the probability distribution of the scenarios, and C is the set of all possible consequences. The meaning and definition of P have been widely discussed in the *Risk Analysis* community, with authors proposing alternative interpretations (Aven, 2010; North, 2010).

Of relevance to us is the wellknown result that Kaplan and Garrick's risk analysis framework yields a multilinear dependence of the risk metric on the basic event probabilities. Therefore, the findings of Proposition 8 apply in a direction of impact setting when a risk analysis is conducted within Kaplan and Garrick's framework.

**FIGURE 7:**
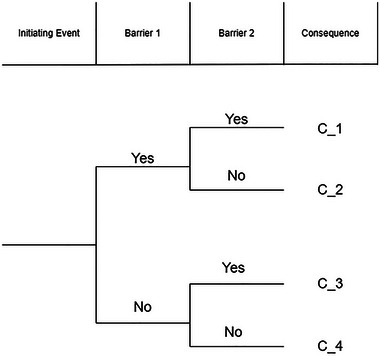
A hypothetical simple event tree.

To illustrate, let us consider the simple hypothetical event tree in Figure 7. The event tree of Figure 7 depicts an initiating event (IE), followed by two possible events (barriers) that can prevent the final undesired consequence (called a top event). If we consider that the end state is binary, the event sequences are judged based on whether they lead to the top event.

For our simple event tree, suppose the top event occurs if the two barriers fail. The top event is then the fourth consequence, $C_{4}$, and we have

(37) $$\begin{array}{c} R=P\left(C_{4}\right)=P\left(\bar{\text{Barrier 1}}\cup \bar{\text{Barrier 1}}\right)=\\ P\left(\bar{\text{Barrier 2}}|\bar{\text{Barrier 1}}\right)P\left(\bar{\text{Barrier 1}}\right), \end{array}$$

where $P(\bar{\text{Barrier 1}})$ is the probability that Barrier 1 fails and $P(\bar{\text{Barrier 2}}|\bar{\text{Barrier 1}})$ is the conditional probability that Barrier 2 fails given that Barrier 1 is not successful. In an input–output form, we have

(38) $$Y=X_{1}X_{2},$$

where Y is the risk metric R, $X_{1}$, and $X_{2}$ are the numerical values, respectively, of $P(\bar{\text{Barrier 1}})$ and $P(\bar{\text{Barrier 2}}|\bar{\text{Barrier 1}})$. Equation (38) is a particular case of Equation (26).

With this premise, we present the investigation of the direction of impact for a realistic example, the probabilistic safety assessment model developed by NASA within its program for the planning of lunar space missions in the early 2000s. We refer to Borgonovo and Smith (2011) for a detailed description of the model. The model decomposes the mission in eight phases: the launch from the Earth and the low Earth orbit of the crew and vehicles for material, which will join in the second phase of the mission and will then insert in the lunar orbit and orbit the moon (third and fourth phases). These phases are followed by the lunar moon orbiting and landing (phases 3 and 4), the lunar mission with the crew performing the required operations (phase 5), the lunar ascent, orbiting, descent towards the Earth and Earth lending covering phases 6–8. The technological part of the mission comprises seven main systems performing the mission's major functions. These are modeled via a probabilistic safety assessment model that takes into account their logical connections. The resulting probabilistic safety assessment (PSA) model contains 150 fault trees and approximately 900 basic events, with a number of minimal cut sets of about 41,446 at a truncation of $10^{-15}$ (Borgonovo & Smith, 2011). Of relevance to us is that the risk metric Y is a multilinear function of the basic event probabilities. Therefore, the assumptions of Proposition 8 hold for this model.

The number of inputs represents a challenge in the direction of impact setting: it is overwhelming for the analyst to inspect the behavior of the output response for 900 inputs. However, a direction of impact analysis is usually performed to gather additional insights on the response of the model to variations in the most important inputs. In our analysis, we benefit from the findings in Borgonovo and Smith (2011) that determine as most important inputs the basic event probabilities $X_{152}$, $X_{143}$, $X_{174}$, $X_{145}$, $X_{177}$, and $X_{179}$.

To examine the response of the risk metric as a function of these inputs, we employ PD functions and ICE plots augmented with the discrepancy and flatness indices introduced in Appendix C. We implement Equations (6), (C.8), and (C.2) numerically in Matlab. The subroutines are available upon request.

**FIGURE 8:**
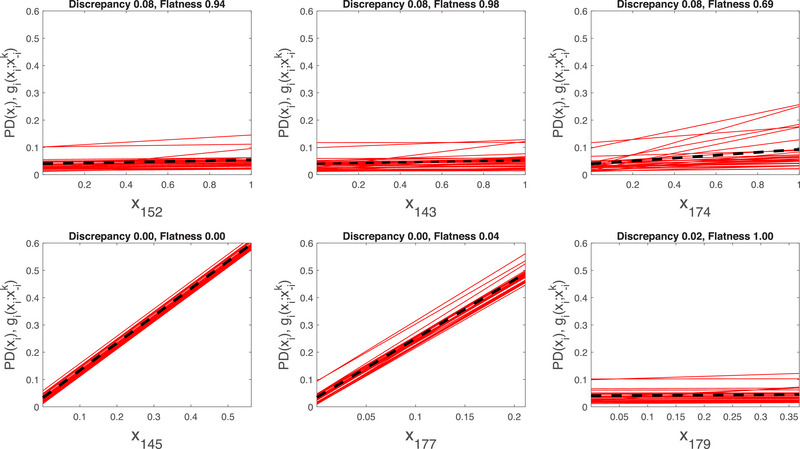
Direction of impact analysis for the NASA space PSA model. Horizontal axis: basic event probability $X_{i}$. Vertical axis: ${\text{PD}}_{i}\left(X_{i}\right)$, $g_{i}\left(x_{i}\right)$ for inputs $X_{152}$, $X_{143}$, $X_{174}$, $X_{145}$, $X_{177}$, and $X_{179}$.

The first panel in Figure 8 shows the PD function (dashed black) and 30 one‐way sensitivity functions (red) obtained when $X_{152}$ varies over its support. All one‐way sensitivity functions and, correspondingly, the PD function are increasing. The flatness index is close to zero confirming that the PD function is not a constant. The discrepancy index is also very low, signaling that the PD function and the one‐way sensitivity functions agree in their indications. We note that one‐way sensitivity functions are not all obtained by adding a constant. Thus, $X_{152}$ is involved in interactions with the other inputs, in line with the results of Borgonovo and Smith (2011). The insights from the second panel for the response of the risk metric as a function of $X_{143}$ are similar. The graph of Y as a function of $X_{174}$ denotes a sharper increase in Y on this input for some one‐way sensitivity functions, signaling that there are some scenarios in which the risk metric becomes increasingly sensitive in this input. The fourth panel shows that all one‐way sensitivity functions and the PD function differ only by a constant. This fact signals that $X_{145}$ is not involved in interactions with the other inputs. Also, the results in Borgonovo and Smith (2011) confirm that Y is additive in this input. From a probabilistic risk analysis viewpoint, this happens, for instance, if the basic event corresponding to $X_{145}$ is in series with the top event and the rare event approximation eliminates the product terms involving the interactions of $X_{145}$ with the remaining basic events.

### 6.2 Computer simulations: SEIR modeling

This section conducts a marginal behavior analysis for a mathematical model for the COVID‐19 pandemic. The simulator is a member of the SEIR family of models (Kermack & McKendrick, 1927), widely used during the COVID pandemic (Currie et al., 2020). The SEIR model we employ follows the works of Peng et al. (2020) in China and Lu and Borgonovo (2023) in Italy and the United States, respectively. The code and data are available from https://github.com/ECheynet/SEIR. The model simulates seven states, to account for the number of individuals susceptible, exposed, infected, recovered, quarantined, deceased, and protected at a given time. The inputs and assigned probability distributions are reported in Table 4. The output of interest is the total number of infected individuals on April 17, 2020, from the beginning of the data series, on February 24, 2020.

**TABLE 4:** Input data for the SEIR model.

Input	$X_{1}=\alpha$	$X_{2}=\beta$,	$X_{3}=\gamma$,	$X_{4}=\delta$
	protection rate	infection rate	average latent time	quarantine rate
Distribution	Uniform	Uniform	Uniform	Uniform
Support	$\alpha^{0}\pm 10\%$	$\beta^{0}\pm 10\%$	$\gamma^{-1}\pm 10\%$	$\delta^{0}\pm 30\%$
**Input**	$X_{5}=I_{0}$	$X_{6}$=Intervention time		
Distribution	Discrete uniform	Discrete uniform		
Support	$I_{0}^{0}\pm 20\%$	08‐Mar‐20+z, z=0,1,⋯,7		

In Lu and Borgonovo (2023), the model is calibrated to Lombardy data of the first months of the pandemic in order to assess the effect of the lockdown time. In Lombardy, the intervention date was March 8, 2020. Intervention time is then assumed as a discrete random variable with 7 days as possible realizations to account for the effect of potential delays in the effectiveness of intervention measures. For the remaining inputs, the reference values $\alpha^{0}$, $\beta^{0},\gamma^{-1,0}$, $\delta^{0}$, and $I_{0}^{0}$ in Table 4 are set by calibrating the model to data after the intervention time in Lombardy. We assume a −0.50 correlation between intervention time and $I_{0}$: the larger the number of initially infected individuals, the sooner intervention measures will be applied. We also assume a 0.75 correlation between infection rate and number of initially infected individuals.

**FIGURE 9:**
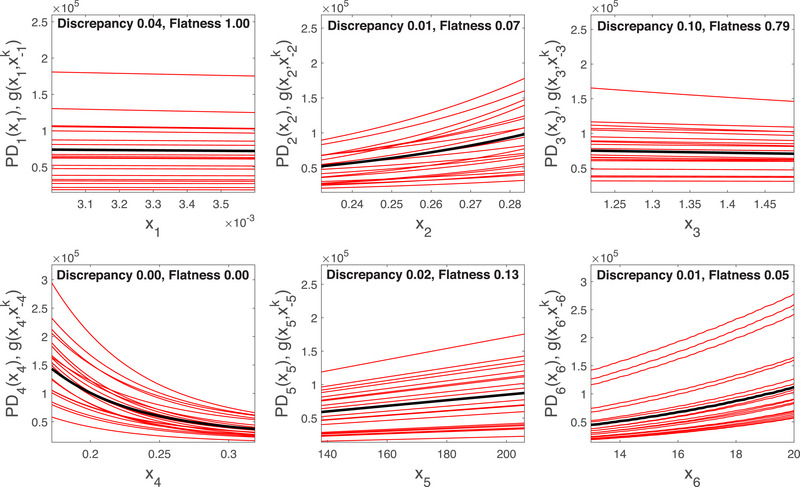
Marginal effects for the six inputs of the SEIR model fitted to Lombardy data in the period February–April 2020. Red lines: ICE functions. Black bold lines: PD functions ${\text{PD}}_{i}\left(x_{i}\right)$.

We propagate uncertainty in the model by generating a Monte Carlo input set from the distributions in Table 4. Correlations are incorporated using the method by Iman and Conover (1982). The simulator is then evaluated in correspondence with the input values; for each realization, the code projects one possible trajectory of the pandemic, and the cumulative number of infected individuals by April 17 is recorded. PD and one‐way sensitivity functions are computed directly with the simulator in the loop. We present results for N=1000 simulations. The overall analysis takes about 20 min on a PC with processor Intel(R), Core(TM) i7‐7700HQ CPU, 2.80 GHz, and 64 GB RAM. Figure 9 displays six ICE/PD plots. The first panel shows the dependence of the cumulative number of infected individuals on the protection rate, $X_{1}$. We record a low discrepancy index ($I_{1}^{\;\text{Discr}}=0.05$) and a flatness index close to 1, indicating that the output is weakly influenced by this input. In contrast, the infection rate, $X_{2}$, has a positive and nonnegligible effect on the simulation output, as one should expect. The average latent time, $X_{3}$, has a negative effect on the simulation output; however, the high flatness index indicates a possibly weak effect. The quarantine rate, $X_{4}$, has a negative effect on the simulation output. Indeed, the higher the efficacy provided by the quarantine, the lower the total number of infected individuals. For this input, we have a discrepancy index close to zero, as all one‐way sensitivity functions are decreasing in $X_{4}$. This suggests that the simulator output might be monotonically decreasing in $X_{4}$. The initial number of infected individuals, $X_{5}=I_{0}$, has a nonnegligible and positive effect on the total number of infected individuals, in accordance with intuition. The practically zero value of the discrepancy index shows a monotonic behavior of the output as a function of $I_{0}$. Also, we record no crossings among one‐way sensitivity functions, suggesting that the output depends monotonically and additively on this input.

The last panel shows the dependence on the total number of infected individuals on the intervention time, $X_{6}$. We record a strong and increasing dependence. Both the discrepancy and the flatness indices are equal to zero, indicating a monotonic behavior of the total number of affected individuals as a function of the intervention time; also, we record no crossings in the one‐way sensitivity functions, which is an indication of a potentially additive behavior of the code as a function of this input. This panel also suggests that an increase in waiting time before intervention would cause the total number of infected individuals to increase rapidly: in expectation, a 7‐day delay amounts to an expected 250% increase in the number of infected individuals.

**FIGURE 10:**
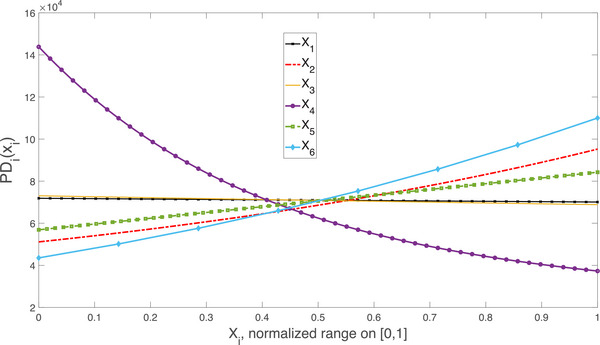
PD spiderplot for the SEIR model with correlated inputs.

Figure 10 reports the PD functions of this case study in a spiderplot, to allow for a more direct visual comparison of the relative strength of the input impacts. The quarantine rate, $X_{4}$, appears to have a stronger impact than the intervention time $X_{6}$, the infection rate $X_{2}$, and the initial number of infected individuals, $X_{5}$. To check these results, we calculated the Kuiper‐based global sensitivity measure of Baucells and Borgonovo (2013) from the available input–output sample, obtaining the same ranking.

Overall, these results confirm that for Lombardy weak or delayed applications of the quarantine and lockdown measures would have led to an even greater increase in the number of infected individuals. It is not unreasonable to expect that such an increased number would have caused additional difficulties in what already was a dramatic situation for the region during the spring of 2020.

## 7 FINAL REMARKS

The creation of quantitative models for risk assessment requires methods for increasing the transparency of the analysis. Assessing the marginal dependence of a risk metric of interest on one or more inputs (direction of impact) becomes a relevant insight. However, the majority of sensitivity analysis studies have focused on the properties of global importance measures within a factor prioritization setting.

We have focused on graphical indicators of marginal dependence, studying and comparing a variety of methods, from classical indicators such as gradients, one‐way sensitivity functions, and correlation coefficients to machine learning methods such as PD functions and ALE plots. For each indicator, we have established whether it delivers insights consistent with the geometric properties of the original input–output mapping. Surprisingly, PD plots are the only global indicators that remain consistent even when inputs are correlated. We have found analytical expressions of all the indicators for the class of multilinear functions, which encompasses several models used in risk analysis.

When coming to recommendations on which indicators to choose, analysts need to integrate a further element with our results: extrapolation risks. The design of PD and one‐way sensitivity functions does not protect from extrapolation issues. Thus, analysts need to carefully evaluate whether such issues are present in their investigations. In contrast, the design of ALE plots mitigates extrapolation risk, making them potentially more suitable for data‐driven applications (Apley & Zhu, 2020). However, the graph of ALE plots may not be consistent with the monotonicity or convexity of the original input–output mapping, especially when features are correlated. Table 5 summarizes these points.

**TABLE 5:** Summary of indicators: Consistency and extrapolation risks.

Case/indicator	One‐way	PD functions	ALE functions	M functions
Independent inputs	Consistent	Consistent	Consistent	Consistent
Dependent inputs	Consistent	Consistent	No	No
Additive recovery	Yes	Yes	Yes	No
Multiplicative recovery	Yes	Yes	No	No
Mitigates extrapolation risk	No	No	Yes	Yes

From Table 5, we see that

- Independent inputs: All indicators are consistent and there is no extrapolation risk, as any combination of the inputs is allowed.
- Dependent inputs: PD functions and one‐way sensitivity functions are monotonicity, Lipschitz, and convexity consistent while consistency for ALE plots and conditional expectation functions is not theoretically guaranteed.
- Extrapolation risk: ALE plots and conditional expectation functions mitigate extrapolation risk, while one‐way and PD functions do not.

While the analysis shows that there is not an overall ideal indicator, we can find two cases in which we can make a safe choice: when inputs are independent (case 1) or when inputs are dependent but extrapolation risk is under control (case 2). Case 1 may be rare in data‐driven applications, although more frequent in the simulation practice, especially in the initial modeling phases. For case 2, when the model equations are derived from first principles or are developed by the analysts using their field knowledge (as in the case of agent‐based simulators), the risk of extrapolation is reduced. In this context, ICE–PD plots become appealing for their consistency which makes their graphs easily readable. However, in a data‐driven context, PD plots are exposed to extrapolation errors. This issue occurs when the machine learning model is asked to make predictions for new points that fall far from the original data. In this context, an analysis based on ALE plots might be preferable (Apley & Zhu, 2020).

The methodological part of our article can be useful to future studies aimed at investigating the properties of alternative (existing or even new) indicators. Further research also concerns the exploration of the relationship between the discrepancy and flatness indices we presented, with the ICE feature impact variable importance of (Yeh & Ngo, 2021) and their integration with the VINE method for the study of one‐way sensitivity functions of Britton (2019). Some preliminary findings can be found in Appendix C.2, however, a full investigation is outside the scope of this article and is part of future work by the authors. Finally, works such as T. Reilly (2000) and Wang et al. (2017) have discussed the implementation of tornado diagrams taking into account input dependences. Integrating these approaches with ALE and PD plots for the direction of change analysis in decision support models (influence diagrams, decision‐tress) is also a future research avenue following the present work.

## CONFLICT OF INTEREST STATEMENT

The authors declare no conflict of interest.

## Figures and Tables

**FIGURE C.1:**
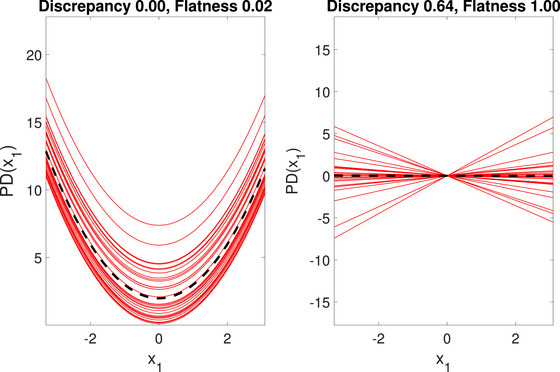
One‐way sensitivity functions (continuous, red) and PD plots (dashed, black bold) for the functions a(X) (left panel) and m(X) (right panel) in Example C.2.

## Footnotes

1 One‐way sensitivity functions $g(x_{i};x_{i}^{s})$ are called individual conditional relationships in Goldstein et al. (2015).

2 Specifically,

(36)
$$r_{1}\left(x_{1}\right)=E\left[g\left(X_{-i}\right)|X_{i}=x_{1}\right]=\frac{x_{1}}{10.42}\exp\left(\frac{-2.285\;7x_{1}^{2}+8.0x_{1}}{2}\right)q\left(x_{1}\right),$$

where $q\left(x_{1}\right)=\int \int \left(x_{2}+x_{3}\right)\exp\left(0.5\left(3.4x_{1}x_{2}+2.3x_{2}^{2}-8.0x_{2}+x_{3}^{2}-2.0x_{3}+9.0\right)\right)dx_{2}dx_{3}$.

3 We displayed one representative one‐way sensitivity function, as they are all constant.

## APPENDIX A PROOFS

Proof of Proposition 1 For simplicity, let us consider the case in which g is increasing. Given the above assumptions, the monotonicity of $r_{i}\left(x_{i}\right)$ can be written as $r_{i}\left(x_{i}+\Delta \right)-r_{i}\left(x_{i}\right)\geq 0$ for every allowed $x_{i}$ and Δ>0. This condition is then equivalent to $E\left[Y|X_{i}=x_{i}+\Delta \right]-E\left[Y|X_{i}=x_{i}\right]\geq 0$. The left‐hand side of the above expression is

(A.1)
$$\begin{array}{c} \int_{X_{-i}}g\left(x_{i}+\Delta ;\;x_{-i}\right)f\left(x_{-i}|X_{i}=x_{i}+\Delta \right)dx_{-i}\\ -\int_{X_{-i}}g\left(x_{i};\;x_{-i}\right)f\left(x_{-i}|X_{i}=x_{i}\right)dx_{-i}, \end{array}$$

which can be equivalently rewritten as

(A.2)
$$\int_{X_{-i}}\;\;\;\left[g\left(x_{i}\;\;+\;\;\Delta ;x_{-i}\right)f\left(x_{-i}|X_{i}=x_{i}\;\;+\;\;\Delta \right)-g\left(x_{i};x_{-i}\right)f\left(x_{-i}|X_{i}=x_{i}\right)\right]dx_{-i}.$$

By the monotonicity of the integral, a sufficient condition for this quantity to be positive(negative) is

(A.3)
$$\begin{array}{ccc} & & g\left(x_{i}+\Delta ;X_{-i}\right)f\left(X_{-i}|X_{i}=x_{i}+\Delta \right)\\ & & \;-g\left(x_{i};X_{-i}\right)f\left(X_{-i}|X_{i}=x_{i}\right)\overset{\left(\leq \right)}{\geq }0 \end{array}$$

for all values of $\left(x_{i}+\Delta ;X_{-i}\right),\left(x_{i};X_{-i}\right)$ in X.□

Proof of Proposition 2 Let us consider the inequality in (8). Then, by multiplying both sides by $f_{X_{-i}}\left(x_{-i}\right)$, we can write

(A.4)
$$g\left(x_{i}+t_{i};x_{-i}\right)f_{X_{-i}}\left(x_{-i}\right)\overset{\left(\leq \right)}{\geq }g\left(x\right)f_{X_{-i}}\left(x_{-i}\right)$$

for all x and $t_{i}>0$. Then, because $g(x_{i};x_{-i})$ is integrable for all $x_{i}\in X_{i}$, by the monotonicity of the integral, we find

(A.5)
$$\int_{X_{-i}}g\left(x_{i}+t_{i};x_{-i}\right)f_{X_{-i}}\left(x_{-i}\right)dx_{-i}\overset{\left(\leq \right)}{\geq }\int_{X_{-i}}g\left(x_{i};x_{-i}\right)f_{X_{-i}}\left(x_{-i}\right)dx_{-i},$$

whence

(A.6)
$${\text{PD}}_{i}\left(x_{i}+t_{i}\right)\overset{\left(\leq \right)}{\geq }{\text{PD}}_{i}\left(x_{i}\right),$$

which is the definition of univariate monotonicity.□

Proof of Corollary 1 If $g=a_{i}\left(x_{i}\right)+a_{-i}\left(x_{-i}\right)$ or $g=m_{i}\left(x_{i}\right)m_{-i}\left(x_{-i}\right)$, then the partial dependence function ${\text{PD}}_{i}\left(x_{i}\right)$ differs from $g(x_{i})$ by an additive or a multiplicative constant. In fact, in the additively separable case, we have

(A.7)
$${\text{PD}}_{i}\left(x_{i}\right)=\int_{X_{-i}}\left(a_{i}\left(x_{i}\right)+a_{-i}\left(x_{-i}\right)\right)f_{X_{-i}}\left(x_{-i}\right)dx_{-i}=a_{i}\left(x_{i}\right)+A_{i},$$

where $A_{i}=\int_{X_{-i}}a_{-i}\left(x_{-i}\right)f_{X_{-i}}\left(x_{-i}\right)dx_{-i}$. Then, in case $PD_{i}\left(x_{i}\right)$ is increasing or decreasing, then $a_{i}\left(x_{i}\right)$ is increasing or decreasing and therefore g is increasing or decreasing. If g is separately multiplicative, we have

(A.8)
$$\begin{array}{c} {\text{PD}}_{i}\left(x_{i}\right)=m_{i}\left(x_{i}\right)\int_{X_{-i}}m_{-i}\left(x_{-i}\right)f_{X_{-i}}\left(x_{-i}\right)dx_{-i}=m_{i}\left(x_{i}\right)M_{i}, \end{array}$$

where $M_{i}=\int_{X_{-i}}m_{-i}\left(x_{-i}\right)f_{X_{-i}}\left(x_{-i}\right)dx_{-i}$. Then, ${\text{PD}}_{i}\left(x_{i}\right)$ is proportional to $m_{i}\left(x_{i}\right)$. Thus, if $M_{i}$ is strictly positive, then g is monotonic with respect to $X_{i}$ with the same monotonicity of ${\text{PD}}_{i}\left(x_{i}\right)$ with respect to $X_{i}$ (if ${\text{PD}}_{i}\left(x_{i}\right)$ is increasing (decreasing) in $X_{i}$, then g is increasing (decreasing)). Conversely, if $M_{i}$ is strictly negative if ${\text{PD}}_{i}\left(x_{i}\right)$ is monotonic, g remains monotonic (increasing or decreasing), but the direction is reversed; that is if ${\text{PD}}_{i}\left(x_{i}\right)$ is increasing, then g is decreasing and vice versa.□

Proof of Proposition 3 Suppose g is increasing in $X_{i}$. Considering first‐order ALE functions, given by

(A.9)
$${\text{ALE}}_{i}\left(x_{i}\right)=\int_{x_{\min,i}}^{x_{i}}E\left[g_{i}^{\prime }\left(X\right)|X_{i}=z_{i}\right]dz_{i},$$

they are the integral of the average of partial derivatives $g_{i}^{\prime }\left(X\right)$ for every value of $X_{i}$. Then, $g_{i}^{\prime }\left(X\right)$ will be consistently positive. Hence, the conditional expectation $E[g_{i}^{\prime }\left(X\right)|X_{i}=z_{i}]$ is positive and, by the monotonicity of the integral, also ${\text{ALE}}_{i}\left(x_{i}\right)$ is positive.□

Proof of Proposition 4 By definition,

(A.10)
$$\begin{array}{ccc} {\text{PD}}_{i}\left(x_{i}^{1}\right)-{\text{PD}}_{i}\left(x_{i}^{0}\right) & = & \int_{X_{-i}}g\left(x_{i}^{1};s\right)dF_{X_{-i}}\left(s\right)\\ & & -\;\int_{X_{-i}}g\left(x_{i}^{0};s\right)dF_{X_{-i}}\left(s\right). \end{array}$$

By the triangle inequality for integrals,

(A.11)
$$\begin{array}{ccc} & & \int_{X_{-i}}\left(g\left(x_{i}^{1};s\right)-g\left(x_{i}^{0};s\right)\right)dF_{X_{-i}}\left(s\right)\\ & & \;\leq \int_{X_{-i}}|g\left(x_{i}^{1};s\right)-g\left(x_{i}^{0};s\right))|dF_{X_{-i}}\left(s\right). \end{array}$$

By the marginal α‐Hölder property of g, we can write

(A.12)
$$\begin{array}{ccc} & & \int_{X_{-i}}|g\left(x_{i}^{1};s\right)-g\left(x_{i}^{0};s\right))|dF_{X_{-i}}\left(s\right)\\ & & \;\leq \int_{X_{-i}}H_{\alpha }{\left|x_{i}^{1}-x_{i}^{0}\right|}^{\alpha }dF_{X_{-i}}\left(s\right). \end{array}$$

Then, noting that the last integral does not involve s, we have

(A.13)
$$\int |g\left(x_{i}^{1};s\right)-g\left(x_{i}^{0};s\right))|dF_{X_{-i}}\left(s\right)\leq H_{\alpha }{\left|x_{i}^{1}-x_{i}^{0}\right|}^{\alpha },$$

which, concatenating the previous inequalities, yields

(A.14)
$$|{\text{PD}}_{i}\left(x_{i}^{1}\right)-{\text{PD}}_{i}\left(x_{i}^{0}\right)|\leq H_{\alpha }{\left|x_{i}^{1}-x_{i}^{0}\right|}^{\alpha },$$

□

Proof of Proposition 5 Consider that g is separately Lipschitz and differentiable with respect to $X_{i}$ on X. Then, we have

(A.15)
$$\lim_{x_{i}^{1}\to x_{i}^{0}}\frac{|g\left(x_{i}^{1};x_{-i}^{0}\right)-g\left(x_{i}^{0};x_{-i}^{0}\right)|}{|x_{i}^{1}-x_{i}^{0}|}=\left|g_{i}^{\prime }\left(x^{0}\right)\right|\leq L.$$

By definition of the ALE plot,

(A.16)
$${\text{ALE}}_{i}\left(x_{i}\right)=\int_{x_{i,\min}}^{x_{i}}\int_{X_{-i}}g_{i}^{\prime }\left(z;x_{-i}\right)f_{X_{i}|X_{-i}}\left(z,x_{-i}\right)dx_{-i}dz,$$

so that by (A.15), we obtain

(A.17)
$$\begin{array}{c} -L\int_{x_{i,\min}}^{x_{i}}\int_{X_{-i}}f_{X_{i}|X_{-i}}\left(z,x_{-i}\right)dx_{-i}dz\leq {\text{ALE}}_{i}\left(x_{i}\right)\\ \leq L\int_{x_{i,\min}}^{x_{i}}\int_{X_{-i}}f_{X_{i}|X_{-i}}\left(z,x_{-i}\right)dx_{-i}dz. \end{array}$$

We define $T_{i}\left(x_{i}\right)$ as the antiderivative of $t_{i}\left(x_{i}\right)=\int_{X_{-i}}f_{X_{i}|X_{-i}}\left(x_{i},x_{-i}\right)dx_{-i}$, and hence $\int_{x_{i,\min}}^{x_{i}}t_{i}\left(z\right)dz=T_{i}\left(x_{i}\right)-T_{i}\left(x_{i,\min}\right)$. We can write

(A.18)
$$-L\cdot \left(T_{i}\left(x_{i}\right)-T_{i}\left(x_{i,\min}\right)\right)\leq {\text{ALE}}_{i}\left(x_{i}\right)\leq L\cdot \left(T_{i}\left(x_{i}\right)-T_{i}\left(x_{i,\min}\right)\right).$$

Then, at $x_{i}^{1}$ and $x_{i}^{0}$, we, respectively, have

(A.19)
$$-L\cdot \left(T_{i}\left(x_{i}^{1}\right)-T_{i}\left(x_{i,\min}\right)\right)\leq {\text{ALE}}_{i}\left(x_{i}^{1}\right)\leq L\cdot \left(T_{i}\left(x_{i}^{1}\right)-T_{i}\left(x_{i,\min}\right)\right),$$

and

(A.20)
$$-L\cdot \left(T_{i}\left(x_{i}^{0}\right)-T_{i}\left(x_{i,\min}\right)\right)\leq {\text{ALE}}_{i}\left(x_{i}^{0}\right)\leq L\cdot \left(T_{i}\left(x_{i}^{0}\right)-T_{i}\left(x_{i,\min}\right)\right),$$

Changing sign to this last equality, we have

(A.21)
$$\begin{array}{ccc} +L\cdot (T_{i}\left(x_{i}^{0}\right)-T_{i}\left(x_{i,\min}\right)) & \geq & -{\text{ALE}}_{i}\left(x_{i}^{0}\right)\\ & \geq & -L\cdot (T_{i}\left(x_{i}^{0}\right)-T_{i}\left(x_{i,\min}\right)), \end{array}$$

which, reordering, yields

(A.22)
$$\begin{array}{c} -L\cdot \left(T_{i}\left(x_{i}^{0}\right)-T_{i}\left(x_{i,\min}\right)+T_{i}\left(x_{i}^{1}\right)-T_{i}\left(x_{i,\min}\right)\right)\\ \leq {\text{ALE}}_{i}\left(x_{i}^{1}\right)-{\text{ALE}}_{i}\left(x_{i}^{0}\right)\\ \leq L\cdot \left(T_{i}\left(x_{i}^{1}\right)-T_{i}\left(x_{i,\min}\right)+T_{i}\left(x_{i}^{0}\right)-T_{i}\left(x_{i,\min}\right)\right). \end{array}$$

□

Proof of Proposition 6 For simplicity, let us start with concavity. If Equation (23) holds, then it holds in particular for pairs of points of the type $x^{1}=\left(x_{i}^{1};x_{-i}^{0}\right)$ and $x^{0}=\left(x_{i}^{0};x_{-i}^{0}\right)$, in which we vary solely the ith component. For these pairs of points, their convex combination is

(A.23)
$$\theta x^{1}+\left(1-\theta \right)x^{0}=\theta \left[\begin{array}{c} x_{1}^{0}\\ \cdots \\ x_{i}^{1}\\ \cdots \\ x_{n}^{0} \end{array}\right]+\left(1-\theta \right)\left[\begin{array}{c} x_{1}^{0}\\ \cdots \\ x_{i}^{0}\\ \cdots \\ x_{n}^{0} \end{array}\right]=\left[\begin{array}{c} x_{1}^{0}\\ \cdots \\ \theta x_{i}^{1}+\left(1-\theta \right)x_{i}^{0}\\ \cdots \\ x_{n}^{0} \end{array}\right].$$

Then, by (A.23), $g[\theta x^{1}+\left(1-\theta \right)x^{0}]$ in (23) specializes into $g(\theta x_{i}^{1}+\left(1-\theta \right)x_{i}^{0};x_{-i}^{0})$, so that the inequality in (23) becomes

(A.24)
$$g\left(\theta x_{i}^{1}+\left(1-\theta \right)x_{i}^{0};x_{-i}^{0}\right)\geq \theta g\left(x_{i}^{1};x_{-i}^{0}\right)+\left(1-\theta \right)g\left(x^{0}\right),\;0\leq \theta \leq 1,$$

which is the definition of concavity for $g(x_{i};x_{-i}^{0})$.□

Proof of Proposition 7 Let θ=α and 1−θ=β. We can rewrite the definition of concavity for g as

(A.25)
$$g\left(\alpha x_{i}^{0}+\beta x_{i}^{1};x_{-i}\right)\geq \alpha g\left(x_{i}^{0};x_{-i}\right)+\beta g\left(x_{i}^{1};x_{-i}\right).$$

Multiplying both sides by $dF_{X_{-i}}\left(x_{-i}\right)$ and recalling that $dF_{X_{-i}}\left(x_{-i}\right)\geq 0$, by the monotonicity of the integral we have

(A.26)
$$\begin{array}{c} \int_{X\setminus X_{i}}g\left(\alpha x_{i}^{0}+\beta x_{i}^{1};x_{-i}\right)dF_{X_{-i}}\left(x_{-i}\right)\\ \geq \alpha \int_{X\setminus X_{i}}g\left(x_{i}^{0};x_{-i}\right)dF_{X_{-i}}\left(x_{-i}\right)+\beta \int_{X\setminus X_{i}}g\left(x_{i}^{1};x_{-i}\right)dF_{X_{-i}}\left(x_{-i}\right). \end{array}$$

Then, by definition of ${\text{PD}}_{i}$, we have

(A.27)
$${\text{PD}}_{i}\left(\alpha x_{i}^{0}+\beta x_{i}^{1}\right)\geq \alpha {\text{PD}}_{i}\left(x_{i}^{0}\right)+\beta {\text{PD}}_{i}\left(x_{i}^{1}\right).$$

□

Proof of Corollary 2 (1) If g is additively separable and Equation (11) holds, then ${\text{PD}}_{i}\left(x_{i}\right)$ differs from $g_{i}\left(x_{i}\right)$ only by an additive constant. Therefore, the convexity of ${\text{PD}}_{i}\left(x_{i}\right)$ implies that $g_{i}\left(x_{i}\right)$ is concave, and thus g is (separately) concave in $X_{i}$. Conversely, if $g_{i}\left(x_{i}\right)$ is concave, then ${\text{PD}}_{i}\left(x_{i}\right)$ is concave because they differ only by an additive constant. (2) The proof in this case is similar, as, in the case g is multiplicatively separable, then ${\text{PD}}_{i}\left(x_{i}\right)$ and $g_{i}\left(x_{i}\right)$ differ only by a multiplicative constant, as by Equation (A.8), we have

(A.28)
$$\begin{array}{c} {\text{PD}}_{i}\left(x_{i}\right)=g_{i}\left(x_{i}\right)M_{i}, \end{array}$$

Then, if $M_{i}>0$ is strictly positive, the concavity of ${\text{PD}}_{i}\left(x_{i}\right)$ and $g_{i}\left(x_{i}\right)$ is the same. Conversely, if $M_{i}<0$ (strictly negative), ${\text{PD}}_{i}\left(x_{i}\right)$ is concave if and only if $g_{i}\left(x_{i}\right)$ is convex.□

Proof of Proposition 8 Letting $Z_{i}$ and $Z_{-i}$ be the subsets of Z that include index i and that exclude it, respectively, then Equation  (26) can be rewritten as

(A.29)
$$g^{ML}\left(x\right)=a_{-i}\left(x_{-i}\right)x_{i}+b_{-i}\left(x_{-i}\right),$$

where $a_{-i}\left(x_{-i}\right)=\sum_{z\in Z_{i}}\beta_{z}\prod_{s\in z,s\neq i}x_{s}$ and $b_{-i}\left(x_{-i}\right)=\sum_{z\in Z_{-i}}\beta_{z}\prod_{s\in z}x_{s}$. Items 1 and 2 follow immediately by (A.29). For the correlation coefficient, consider the numerator of $\rho_{Y\;X_{i}}$, $Cov(Y,X_{i})$. By definition of covariance, we have

(A.30)
$$Cov\left(Y,X_{i}\right)=E\left[\left(X_{i}\right)\left(A_{i}X_{i}+B_{i}\right)\right]-{\bar{x}}_{i}\bar{y},$$

where we have set $\bar{y}=E\left[Y\right]$, ${\bar{x}}_{i}=E\left[X_{i}\right]$, $B_{i}=b_{-i}\left(X_{-i}\right)$, $A_{i}=a_{-i}\left(X_{-i}\right)$

(A.31)
$$\begin{array}{cc} \rho_{Y\;X_{i}} & =E\left[\left(X_{i}\right)\left(A_{i}X_{i}+B_{i}\right)\right]-{\bar{x}}_{i}\bar{y}\\ & ==E\left[\left(X_{i}\right)\left(A_{i}X_{i}+B_{i}\right)\right]-{\bar{x}}_{i}E\left[A_{i}X_{i}+B_{i}\right]\\ & =E\left[A_{i}X_{i}^{2}+B_{i}X_{i}\right]-{\bar{x}}_{i}E\left[A_{i}X_{i}+B_{i}\right]\\ & =E\left[a_{-i}\left(X_{-i}\right)X_{i}^{2}+b_{-i}\left(X_{-i}\right)X_{i}\right]-{\bar{x}}_{i}E\left[a_{-i}\left(X_{-i}\right)X_{i}+b_{-i}\left(X_{-i}\right)\right]. \end{array}$$

For item 3, note that

(A.32)
$$\begin{array}{c} {\bar{a}}_{-i}=\int_{X_{-i}}a_{-i}\left(x_{-i}\right)f_{X_{-i}}\left(x_{-i}\right)dx_{-i}\\ =\int_{X_{-i}}a_{-i}\left(x_{-i}\right)\int_{X_{i}}f_{X}\left(x\right)dx_{i}dx_{-i}=\int_{X_{-i}}\int_{X_{i}}a_{-i}\left(x_{-i}\right)f_{X}\left(x\right)dx_{i}dx_{-i}\\ =\int_{X}a_{-i}\left(x_{-i}\right)f_{X}\left(x\right)dx=E\left[a_{-i}\left(X_{-i}\right)\right], \end{array}$$

where we have used the fact that

(A.33)
$$f_{X_{-i}}\left(x_{-i}\right)=\int_{X_{i}}f_{X}\left(x\right)dx_{i}.$$

Because $a_{-i}\left(X_{-i}\right)=g_{i}^{\prime }\left(X\right)$, we also have

(A.34)
$$\begin{array}{c} r_{i}\left(x_{i}\right)=\int_{X_{-i}}\left[a_{-i}\left(x_{-i}\right)x_{i}+b_{-i}\left(x_{-i}\right)\right]dF_{X_{-i}}\left(x_{-i}\right)=E\left[x_{i}g_{i}^{\prime }\left(X\right)|X_{i}=x_{i}\right]\\ +E[b_{-i}\left(X_{-i}\right)|X_{i}=x_{i}], \end{array}$$

and

(A.35)
$$\begin{array}{cc} {\text{ALE}}_{i}\left(x_{i}\right) & =\int_{x_{\min,i}}^{x_{i}}\int \cdots \int g_{i}^{\prime }\left(X\right)dF_{X_{-i}|X_{i}}\left(X_{-i};t\right)\mathrm{dt}\\ & =\int_{x_{\min,i}}^{x_{i}}\int \cdots \int a_{-i}\left(X_{-i}\right)dF_{X_{-i}|X_{i}}\left(X_{-i},t\right)\mathrm{dt}\\ & =\int_{x_{\min,i}}^{x_{i}}E\left[a_{-i}\left(X_{-i}\right)|X_{i}=t\right]\mathrm{dt} \end{array}$$

and

(A.36)
$${\bar{a}}_{-i}=E\left[g_{i}^{\prime }\left(X\right)\right].$$

For the third point, if $X_{i}$ is independent of $X_{-i}$, we have

(A.37)
$${\text{ALE}}_{i}\left(x_{i}\right)=E\left[a_{-i}\left(X_{-i}\right)\right]\left(x_{i}-x_{\min,i}\right)={\bar{a}}_{-i}\left(x_{i}-x_{\min,i}\right),$$

and

(A.38)
$$r_{i}\left(x_{i}\right)=x_{i}E\left[g_{i}^{\prime }\left(X\right)\right]+E\left[b_{-i}\left(X_{-i}\right)\right]={\bar{a}}_{-i}x_{i}+{\bar{b}}_{-i}={\text{PD}}_{i}\left(x_{i}\right),$$

and

(A.39)
$$\begin{array}{cc} \rho_{Y\;X_{i}} & =E\left[a_{-i}\left(X_{-i}\right)\right]E\left[X_{i}^{2}\right]+E\left[b_{-i}\left(X_{-i}\right)\right]{\bar{x}}_{i}\\ & \;-{\bar{x}}_{i}^{2}E\left[a_{-i}\left(X_{-i}\right)\right]-{\bar{x}}_{i}E\left[b_{-i}\left(X_{-i}\right)\right]\\ & =E\left[a_{-i}\left(X_{-i}\right)\right]\left(E\left[X_{i}^{2}\right]-{\bar{x}}_{i}^{2}\right)={\bar{a}}_{-i}\sigma_{i}^{2}. \end{array}$$

□

## APPENDIX B NO RELATIONSHIP BETWEEN THE LIPSCHITZ PROPERTY AND $R_{I}\left(X_{I}\right)$

The reason for the lack of relationship is related to the fact that for conditional expectation functions it is not possible to exploit the additivity of integrals in the same way as it is possible for PD functions. In particular, the step

(B.1)
$$\begin{array}{c} \int_{X_{-i}}g\left(x_{i}^{1};s\right)dF_{X_{-i}}\left(s\right)-\int_{X_{-i}}g\left(x_{i}^{0};s\right)dF_{X_{-i}}\left(s\right)\\ =\int_{X_{-i}}g\left(x_{i}^{1};s\right)-g\left(x_{i}^{0};s\right)dF_{X_{-i}}\left(s\right), \end{array}$$

where we factor $dF_{X_{-i}}\left(s\right)$ out cannot be performed. In fact, we have

(B.2)
$$\begin{array}{ccc} r_{i}\left(x_{i}^{1}\right)-r_{i}\left(x_{i}^{0}\right) & = & \int_{X_{-i}}\;\;g\left(x_{i}^{1};s\right)dF_{X_{-i}|X_{i}}\left(s;x_{i}^{1}\right)\\ & & -\int_{X_{-i}}\;\;g\left(x_{i}^{0};s\right)dF_{X_{-i}}\left(s;x_{i}^{0}\right), \end{array}$$

and, because $dF_{X_{-i}|X_{i}}\left(s;x_{i}^{1}\right)$ depends on $X_{i}$ we cannot factor it out as a common term in the difference as in Equation (B.1). This then prevents one from linking the difference $r_{i}\left(x_{i}^{1}\right)-r_{i}\left(x_{i}^{0}\right)$ to the Lipschitz property of g, unless additional assumptions are added, such as independence.

## APPENDIX C AIDING THE INTERPRETATION OF ICE PLOTS: A DISCREPANCY AND A FLATNESS INDEX

This appendix is divided into two parts. In the first part, we provide the definition and properties of the indices. In the second part, we discuss numerical aspects and provide connections with indices previously defined in the literature.

### C.1. Definitions and properties

Example 13 showcases an instance in which PD and one‐way sensitivity functions provide very different indications.

To facilitate the reading of the graph, we propose an index that provides a synthetic indication of the discrepancy. The ideal index would be zero when indications coming from one‐way and PD functions coincide and would take the value 1 when information is always opposite. To introduce such an index, consider the random ratio

(C.1)
$$W_{i}=\frac{g\left(X_{i}+\Delta ;X_{-i}\right)-g\left(X\right)}{{\text{PD}}_{i}\left(X_{i}+\Delta \right)-{\text{PD}}_{i}\left(X_{i}\right)},$$

with $X_{i}+\Delta ,X_{-i},X$ belonging to X and with ${\text{PD}}_{i}\left(X_{i}+\Delta \right)-{\text{PD}}_{i}\left(X_{i}\right)\neq 0$ (The case ${\text{PD}}_{i}\left(X_{i}+\Delta \right)-{\text{PD}}_{i}\left(X_{i}\right)=0$ is investigated later on). The ratio $W_{i}$ in turn equals the ratio between $\frac{g\left(X_{i}+\Delta ;X_{-i}\right)-g\left(X\right)}{\Delta }$, the Newton quotient centered at X and computed along the one‐way sensitivity function $g(X_{i};X_{-i})$, and $\frac{{\text{PD}}_{i}\left(X_{i}+\Delta \right)-{\text{PD}}_{i}\left(X_{i}\right)}{\Delta }$, the Newton quotient computed along the partial dependence function ${\text{PD}}_{i}\left(X_{i}\right)$. If these quotients have the same sign, then the one‐way sensitivity function $g(X_{i}+\Delta ;X_{-i})$ and the partial dependence function ${\text{PD}}_{i}\left(X_{i}\right)$ provide equivalent indications. That is, if the sign of $W_{i}$ is positive, then the indication is concordant, if the sign is negative, the indication is discordant. We define the following *discrepancy index*,

(C.2)
$$I_{i}^{\text{Discr}}=\frac{1-E[\text{sgn}\left(W_{i}\right)]}{2}.$$

Proposition C.1
($I_{i}^{\text{Discr}}$: some properties) We have that $0\leq I_{i}^{\text{Discr}}\leq 1$, for every g, $\left(X,B\left(X\right),P_{X}\right)$ and i=1,2,⋯,n. If g is additive or separately monotonic in $X_{i}$ then $I_{i}=0$.

Proof of Proposition C.1 First part 1: the quantity $\text{sgn}(W_{i})$ is a random quantity equal to 1 or −1. Thus, the expectation of such quantity cannot exceed 1 or −1. In the case $\text{sgn}(W_{i})=-1$ for any realization of $X_{i}$, we would have $I_{i}^{\text{Discr}}=\frac{1-(-1)}{2}=1$. Conversely, if $\text{sgn}(W_{i})=1$ for all realizations of $X_{i}$, then $I_{i}^{\text{Discr}}=0$. Second part: this part contains two statements: the first regards separately additive mappings and the second monotone mappings. If g(x) is separately additive then Equations (11) and (A.7) hold. Then, we have

(C.3)
$$\begin{array}{cc} W_{i} & =\frac{a_{i}\left(X_{i}+\Delta \right)+A_{-i}\left(X_{-i}\right)-t_{i}\left(X_{i}\right)-A_{-i}\left(X_{-i}\right)}{a_{i}\left(X_{i}+\Delta \right)+A_{i}+a_{i}\left(X_{i}\right)-A_{i}}\\ & =\frac{a_{i}\left(X_{i}+\Delta \right)-a_{i}\left(X_{i}\right)}{a_{i}\left(X_{i}+\Delta \right)-a_{i}\left(X_{i}\right)}=1 \end{array}$$

for all $X_{i}$ and, therefore $\text{sgn}(W_{i})=1$ for all $X_{i}$. Hence, $I_{i}^{\text{Discr}}=\frac{1-1}{2}=0$. If g is separately monotonic, we have the following. We have proven that one‐way sensitivity functions are monotonicity and concavity preserving. Therefore, if g is increasing (decreasing) then $g\left(X_{i}+\Delta ;X_{-i}\right)-g\left(X\right)/\Delta$ is positive (negative) for all $X_{i}$ and Δ.□

Thus, if the indications of PD functions and one‐way sensitivity functions are always consistent, $W_{i}$ is always positive, and $I_{i}^{\text{Discr}}=0$ (no discrepancy). Small values of $I_{i}^{\;\text{Discr}\;}$ indicate high agreement between one‐way and PD functions. Conversely, a value $I_{i}^{\text{Discr}}=1$ suggests that the indications are different with probability 1. The second part of Proposition C.1 addresses two sufficient conditions under which $g(x_{i};x_{-i})$ always agree: the case in which g is additive or separately monotonic. As a consequence, values of $I_{i}^{\text{Discr}}$ different from zero imply that g cannot be additive or monotonic. To illustrate $I_{i}^{\text{Discr}}$ further, we discuss its empirical version in Appendix C.2, where we also present the connection of C.2 with other indices introduced in the literature and a novel connection with a recently introduced importance measure.
Example C.1 Consider the mappings $a\left(X\right)=X_{1}^{2}+X_{2}^{2}+X_{3}^{2}$, and $m\left(X\right)=X_{1}X_{2}X_{3}$, with $X_{1}$, $X_{2}$, and $X_{3}$ uniformly and independently distributed on $X={\left[0,1\right]}^{3}$. Then, both mappings are separately increasing in $X_{i}$. The PD functions are

(C.4)
$$\begin{array}{cccc} {\text{PD}}_{i}^{a}\left(x_{i}\right)=x_{i}^{2}+\frac{2}{3} & \;\text{and}\; & {\text{PD}}_{i}^{m}\left(x_{i}\right)=\frac{x_{i}}{4}, & \;\text{for}\;i=1,2,3, \end{array}$$

and report exactly the marginal behavior of the two input–output mappings. We have

1. For the additive mapping,
  (C.5)
  $$W_{1}=\frac{a\left(x_{1}+\Delta ,X_{2},X_{3}\right)-a\left(x\right)}{{\text{PD}}_{1}^{a}\left(x_{1}+\Delta \right)-{\text{PD}}_{1}^{a}\left(x_{1}\right)}=\frac{\Delta \left(2x_{1}+\Delta \right)}{\Delta \left(2x_{1}+\Delta \right)}=1.$$
  Thus, one‐way sensitivity and PD functions deliver consistent information at any $X_{1}$ and $X_{1}+\Delta$ in the domain, leading to $I_{i}^{\text{Discr}}=0$.
2. For the multiplicative mapping m(X),
  (C.6)
  $$W_{1}=\frac{m\left(x_{1}+\Delta ,X_{2},X_{3}\right)-m\left(X\right)}{{\text{PD}}_{1}^{m}\left(x_{1}+\Delta \right)-{\text{PD}}_{1}^{m}\left(x_{1}\right)}=\frac{X_{2}X_{3}}{E[X_{2}X_{3}]}.$$
  On ${\left[0,1\right]}^{3}$, m(X) is separately monotonic in any of its variables and thus $I_{i}^{\text{Discr}}=0$. Note also that on this domain $E[X_{2}X_{3}]$ is positive and $X_{2}$ and $X_{3}$ are positive with probability 1.
3. For the mapping in Example 13, we have that
  (C.7)
  $$\begin{array}{cc} W_{1} & =\frac{\left(\left(x_{1}+\Delta \right)X_{2}+\left(x_{1}+\Delta \right)X_{3}\right)-\left(X_{1}X_{2}+X_{1}X_{3}\right)}{2\left(x_{1}+\Delta \right)-2x_{1}}\\ & =\frac{1}{2}\left(X_{2}+X_{3}\right). \end{array}$$
  The input–output mapping is neither monotonic nor additive. The sign of $W_{1}$ depends on the point at which we are calculating the one‐way sensitivity functions. Thus, $g(x_{i};X_{-i})$ and ${\text{PD}}_{i}\left(x_{i}\right)$ do not always deliver concordant information for this mapping.

Note that for a separately multiplicative mapping, we have $W_{i}=\frac{\prod_{j=1,j\neq i}^{n}X_{j}}{E[\prod_{j=1,j\neq i}^{n}X_{j}]}$. Thus, $W_{i}$ is always positive if at any point $X_{-i}$, the product $\prod_{j=1,j\neq i}^{n}X_{j}$ is concordant with $E[\prod_{j=1,j\neq i}^{n}X_{j}]$. This is the case when the mapping is monotonic.
Example C.2 Consider the additive and multiplicative functions in Example C.1, but now with $X_{1}$, $X_{2}$, and $X_{3}$ independently and standard normally distributed. The respective ICE plots are given in the left and right panels of Figure C.1. Each plot reports 100 one‐way sensitivity functions. For the additive function, the estimated $I_{i}^{\text{Discr}}$ is zero for all variables. Note that we have concordance even if the mapping is not separately monotonic. In contrast, for the multiplicative function m(x) in Example C.2, we record $I_{i}^{\text{Discr}}=1$. The input–output mapping is not monotonic, one‐way sensitivity functions are linear, with positive and negative slopes, and the resulting PD functions are flat.

Example C.2 demonstrates two extreme situations. In the left panel of Figure C.1, the average information provided by PD functions accurately summarizes the local information of one‐way sensitivity functions. In contrast, in the right panel of Figure C.1, PD functions are not informative (they are flat), and one needs to resort to one‐way sensitivity functions to understand the dependence of g on $X_{2}$. This is the result of the flat expectation effect. Note that the flatness of ${\text{PD}}_{i}\left(x_{i}\right)$ makes the denominator in Equation (C.1) vanish so that $I_{i}^{\text{Discr}}$ is not defined. We excluded this case in the definition of $I_{i}^{\text{Discr}}$.

In order to account for the flat expectation effect, we introduce a flatness index defined as follows. Consider an ordered sequence of realizations of $X_{i}$, $x_{i}^{1},x_{i}^{2},\cdots ,x_{i}^{k}$ (this sequence can be obtained by sorting all realizations of $X_{i}$ or it can just be an ordered subset), with k=1,2,⋯,R and R≤N. Also, introduce a counter initialized at 0. Then, consider the difference $|{\text{PD}}_{i}\left(x_{i}^{k+1}\right)-{\text{PD}}_{i}\left(x_{i}^{k}\right)|$. If this difference is zero, we add 1 to the counter; conversely, we proceed to the next k. The ratio between the number of times at which the flatness condition is encountered and R (the total number of times it is computed) is then an indicator of flatness. In fact, if a function is constant the final value of the counter would be equal to R and the index would be equal to unity (complete flatness). Conversely, if a function is strictly monotonic, the counter would be empty and the flatness index would equal zero. 4 A more refined version of the index could consist of taking the difference $|{\text{PD}}_{i}\left(x_{i}^{k+1}\right)-{\text{PD}}_{i}\left(x_{i}^{k}\right)|$ for all pairs of points in $\left(x_{i}^{1},x_{i}^{2},\cdots ,x_{i}^{k}\right)$. However, this intuition needs to be slightly modified to account for numerical stability. That is, we may find a very small but still not zero value of $|{\text{PD}}_{i}\left(x_{i}^{k+1}\right)-{\text{PD}}_{i}\left(x_{i}^{k}\right)|$ because of numerical noise (approximations and rounding). To overcome this problem, one can establish a threshold ($\varepsilon_{flat}$), such that $|{\text{PD}}_{i}\left(x_{i}^{k+1}\right)-{\text{PD}}_{i}\left(x_{i}^{k}\right)|$ is deemed zero if $|{\text{PD}}_{i}\left(x_{i}^{k+1}\right)-{\text{PD}}_{i}\left(x_{i}^{k}\right)|<\varepsilon_{flat}\left|{\text{PD}}_{i}\left(x_{i}^{k}\right)\right|$. Then, we define a *flatness index* as

(C.8)
$$I_{i}^{\text{Flat}}=\frac{\#\{|{\text{PD}}_{i}\left(x_{i}^{k+1}\right)-{\text{PD}}_{i}\left(x_{i}^{k}\right)|<\varepsilon_{flat}|{\text{PD}}_{i}\left(x_{i}^{k}\right)|\}}{R},$$

where #{·} is the counting function that returns the number of times the argument is met. This is an empirical index that complements $I_{i}^{\text{Discr}}$. In fact, if $I_{i}^{\text{Flat}}\approx 1$, then the analyst has an indication that the behavior of the PD function tends to be flat over the interval of interest. A high value of $I_{i}^{\text{Flat}}$ together with a high value of $I_{i}^{\text{Discr}}$ is indicative of the flat expectation effect. That is, one‐way sensitivity functions indicate a positive and negative dependence, while their average (the PD function) is zero and does not provide meaningful information. Conversely, the case $I_{i}^{\text{Discr}}=0$ and $I_{i}^{\;\text{Flat}}>0$ indicates an additive or monotonic input–output mapping. Finally, the case $I_{i}^{\text{Discr}}=0$ and $I_{i}^{\text{Flat}}=0$ indicates that g has a weak (if not absent) dependence on $X_{i}$.

### C.2. Further details on the discrepancy index and connections with other indices

We discuss additional details on the computation of $I_{i}^{\text{Discr}}$ in Equation (C.2) and the connection with two important measures.

Let K be the number of one‐way sensitivity functions and let $\left[x_{i}^{1},x_{i}^{2},\cdots ,x_{i}^{A}\right]$ be a grid of values for input $X_{i}$. Then, for a∈[1,2,⋯,A] and k=1,2,⋯,K, we can write

(C.9)
$$w_{i}^{\left(k,a\right)}=\frac{g\left(x_{i}^{a};x_{-i}^{k}\right)-g\left(x_{i}^{a-1};x_{-i}^{k}\right)}{{\text{PD}}_{i}\left(x_{i}^{a}\right)-{\text{PD}}_{i}\left(x_{i}^{a-1}\right)},$$

where $g_{i}\left(x_{i}^{a};x_{-i}^{k}\right)$ and $g_{i}\left(x_{i}^{a-1};x_{-i}^{k}\right)$ are the values of the kth one‐way sensitivity function at $\left(x_{i}^{a};x_{-i}^{k}\right)$ and $\left(x_{i}^{a-1};x_{-i}^{k}\right)$, respectively, and ${\text{PD}}_{i}\left(x_{i}^{a}\right)$ and ${\text{PD}}_{i}\left(x_{i}^{a-1}\right)$ are the values of the PD function at $x_{i}^{a}$ and $x_{i}^{a-1}$. (For notation simplicity, for the moment, we consider only differences between adjacent points $x_{i}^{a}$ and $x_{i}^{a-1}$ but one can in principle consider any pair of points on the grid. We relax this later in this appendix). Thus, $w_{i}^{\left(k,a\right)}$ explore a ratio of differences between the values of the one‐way sensitivity functions and the PD functions across several one‐way sensitivity functions and along each one‐way sensitivity function. Note that if we multiply and divide the right‐hand side in Equation (C.9) by $\left(x_{i}^{a}-x_{i}^{a-1}\right)$ we obtain

(C.10)
$$w_{i}^{\left(k,a\right)}=\frac{g\left(x_{i}^{a};x_{-i}^{k}\right)-g\left(x_{i}^{a-1};x_{-i}^{k}\right)/\left(x_{i}^{a}-x_{i}^{a-1}\right)}{{\text{PD}}_{i}\left(x_{i}^{a}\right)-{\text{PD}}_{i}\left(x_{i}^{a-1}\right)/\left(x_{i}^{a}-x_{i}^{a-1}\right)},$$

which is the ratio between a Newton ratio on the kth one‐way sensitivity function and on the PD function. Thus, a sign of $w_{i}^{\left(k,a\right)}$ equal to unity implies that the signs of the Newton ratios calculated on the one‐way sensitivity and the PD function are the same.

Formally, we have

(C.11)
$$\text{sgn}\left(\;w_{i}^{\left(k,a\right)}\right)\;=\;\;\left\{\;\;\begin{array}{ccc} 1\;\; & \;\;\text{if}\; & \left(g\left(x_{i}^{a};\;x_{-i}^{k}\right)\;-\;g\left(x_{i}^{a-1};\;x_{-i}^{k}\right)\right)\left({\text{PD}}_{i}\left(x_{i}^{a}\right)\;-\;{\text{PD}}_{i}\left(x_{i}^{a-1}\right)\right)\;>\;0\\ -1\;\; & & \text{otherwise.} \end{array}\right.$$

We obtain an estimate of $I_{i}^{\text{Discr}}$ by taking the average of all the changes:

(C.12)
$${\hat{I}}_{i}^{\text{Discr}}=\frac{1}{2}-\sum_{k=1}^{K}\sum_{a=2}^{A}\frac{\text{sgn}(w_{i}^{\left(k,a\right)})}{2K(A-1)}.$$

More generally, we can also compute the differences for all nonidentical points $\left(x_{i}^{a^{\prime }};x_{-i}^{k}\right)$ and $\left(x_{i}^{a};x_{-i}^{k}\right)$, obtaining

(C.13)
$$w_{i}^{\left(k,a^{\prime },a\right)}=\frac{g\left(x_{i}^{a^{\prime }};x_{-i}^{k}\right)-g\left(x_{i}^{a};x_{-i}^{k}\right)}{{\text{PD}}_{i}\left(x_{i}^{a^{\prime }}\right)-{\text{PD}}_{i}\left(x_{i}^{a}\right)},$$

where $a,a^{\prime }\in \left[1,2,\cdots ,A\right]$, $a\neq a^{\prime }$ and define ${\hat{I}}_{i}^{\text{Discr}}$ based on the sign of $w_{i}^{\left(k,a^{\prime },a\right)}$.

We observe two connections between the design that allow us to estimate ${\hat{I}}_{i}^{\text{Discr}}$ and two feature importance measures. The first is ICE feature impact of Yeh and Ngo (2021), defined as,

$$FI_{i}=\frac{\sigma_{i}}{K(A-1)}\sum_{k=1}^{K}\sum_{a=2}^{A}\left|\frac{g\left(x_{i}^{a};x_{-i}^{k}\right)-g\left(x_{i}^{a-1};x_{-i}^{k}\right)}{x_{i}^{a}-x_{i}^{a-1}}\right|,$$

where $\sigma_{i}$ is the standard deviation of $X_{i}$. The feature impact of $X_{i}$ is then its standard deviation multiplied by the average of the absolute difference quotients $|g\left(x_{i}^{a};x_{-i}^{k}\right)-g\left(x_{i}^{a-1};x_{-i}^{k}\right)/\left(x_{i}^{a}-x_{i}^{a-1}\right)|$, which are again obtained from the same set of model evaluations needed to find ${\hat{I}}_{i}^{\text{Discr}}$. (They form the numerator in Equation (C.10)).

The second is an original connection we sketch here. Consider starting from $w_{i}^{\left(k,a^{\prime },a\right)}$ and taking the average of the squared finite differences in the numerator of Equation (C.9). We obtain the estimate

(C.14)
$${\hat{\psi }}_{i}=\sum_{k=1}^{K}\sum_{a=2}^{A}\sum_{a^{\prime }\neq a}\frac{{\left(g\left(x_{i}^{a^{\prime }};x_{-i}^{k}\right)-g\left(x_{i}^{a};x_{-i}^{k}\right)\right)}^{2}}{2K(A-1)(A-2)}.$$

The quantity ${\hat{\tau }}_{i}^{2}$ is an estimate of the $\psi_{i}$ index defined in Equation (8) of Verdinelli and Wasserman (2023). The population version of ${\hat{\psi }}_{i}$ is

$$\psi_{i}=\frac{1}{2}\int \int {\left(g\left(x_{i}^{\prime };x_{-i}\right)-g\left(x_{i};x_{-i}\right)\right)}^{2}dF\left(x\right)dF_{i}\left(x_{i}^{\prime }\right).$$

When the inputs are independent, $\psi_{i}$ coincides with the total index of $X_{i}$, as a result in Jansen (1999). Concisely, we recall that

- total indices are defined as the expected portion of the variance of Y=g(X) that remains after we fix all inputs but $X_{i}$ (Saltelli & Tarantola, 2002)
- under independence, they coincide with the contribution to the variance of Y from $X_{i}$, including its individual contribution and all its interactions with the remaining terms (Homma & Saltelli, 1996).

Studying the estimation of $FI_{i}$ and $\psi_{i}$ in association with ICE plots goes beyond the scope of the present work but represents an interesting path for future research.

## APPENDIX D CALCULATIONS FOR THE ALE FUNCTION IN EXAMPLE 11

Given that $X_{1}$ and $X_{2}$ are bivariate normal random variables, the conditional distribution of $X_{2}$ given $X_{1}$ is a normal distribution with mean $\mu_{2}+\frac{\sigma_{2}}{\sigma_{1}}\rho \left(t-\mu_{1}\right)$ and variance $\left(1-\rho^{2}\right)\sigma_{1}^{2}$. Let us denote the corresponding density with $\phi (\mu_{2}+\frac{\sigma_{2}}{\sigma_{1}}\rho \left(t-\mu_{1}\right),\left(1-\rho^{2}\right)\sigma_{1}^{2})$. Given $y=x_{1}x_{2}$, we have $\frac{\partial y}{\partial x_{1}}=x_{2}$. Inserting in the definition of the ALE function, we have

(D.1)
$$\begin{array}{ccc} ALE_{1}\left(x_{1}\right) & = & \int_{x_{1}{,}_{\min}}^{x_{1}}dt\int_{-\infty }^{\infty }x_{2}\phi \left(\mu_{2}+\frac{\sigma_{2}}{\sigma_{1}}\rho \left(t-\mu_{1}\right),\left(1-\rho^{2}\right)\sigma_{1}^{2}\right)dx_{2}\\ & = & \int_{x_{1}{,}_{\min}}^{x_{1}}\left[\mu_{2}+\frac{\sigma_{2}}{\sigma_{1}}\rho \left(t-\mu_{1}\right)\right]dt=\left[\mu_{2}t+\frac{1}{2}\frac{\sigma_{2}}{\sigma_{1}}\rho \left(t^{2}-\mu_{1}t\right)\right]{|}_{x_{1}{,}_{\min}}^{x_{1}}\\ & = & \left[\left(\mu_{2}-\frac{\sigma_{2}}{\sigma_{1}}\rho \mu_{1}\right)t+\frac{1}{2}\frac{\sigma_{2}}{\sigma_{1}}\rho t^{2}\right]{|}_{x_{1}{,}_{\min}}^{x_{1}}\\ & = & \left(\mu_{2}-\frac{\sigma_{2}}{\sigma_{1}}\rho \mu_{1}\right)\left(x_{1}-x_{1,\min}\right)+\frac{1}{2}\frac{\sigma_{2}}{\sigma_{1}}\rho \left(x_{1}^{2}-x_{1}^{2}{,}_{\min}\right) \end{array}.$$

This equation shows that the $ALE_{1}\left(x_{1}\right)$ depends on the choice of $x_{1,\min}$, and it would be undefined if we chose as $x_{1,\min}$ the left extreme of the normal distribution (−∞). Also, for ρ≠0, we have a quadratic dependence on $x_{1}$, which differs from the true input–output dependence, which is linear in $x_{1}$. Setting $\sigma_{1}=\sigma_{2}=\mu_{1}=\mu_{2}=1$, we obtain

(D.2)
$$ALE_{1}\left(x_{1}\right)=\left(1-\rho \right)\left(x_{1}-x_{1,\min}\right)+\frac{\rho }{2}\left(x_{1}^{2}-x_{1}^{2}{,}_{\min}\right).$$
